# Modeling and targeting the hostile physicochemical niche in bone metastasis: from experimental platforms to niche-directed therapy

**DOI:** 10.3389/fcell.2026.1894171

**Published:** 2026-07-29

**Authors:** Mohamad Bakir, Abdul Rahman Alkhatib, Baraa Helal, Mohammad Khaldoun Al Masri, Alhomam Dabaliz, Khalid Said Mohammad

**Affiliations:** 1 Department of Medicine, College of Medicine, Alfaisal University, Riyadh, Saudi Arabia; 2 Department of Clinical Skills, College of Medicine, Alfaisal University, Riyadh, Saudi Arabia; 3 Department of Anatomy, College of Medicine, Alfaisal University, Riyadh, Saudi Arabia

**Keywords:** bone metastasis, bone-on-chip, MPS, niche-directed therapy, physicochemical niche, therapy resistance

## Abstract

Cancer metastasis to bone is shaped not only by tumor-stromal crosstalk but also by a hostile physicochemical niche that promotes disease persistence and therapeutic failure. Hypoxia, acidosis, mineralized matrix architecture, altered mechanics, interstitial pressure, impaired perfusion, and limited drug transport influence metastatic seeding, dormancy, reactivation, remodeling, pain, and treatment response. Conventional two-dimensional cultures and many simplified three-dimensional systems reproduce only selected features of this environment, often overestimating therapeutic efficacy. This review examines experimental platforms designed to model bone metastasis with greater biological and biophysical fidelity, including mineralized scaffolds, multicellular co-cultures, *ex vivo* bone explants, organoids, bone-on-chip and microphysiological systems (MPS), animal models, and computational or hybrid approaches. Rather than ranking models by complexity, we propose a fit-for-purpose framework in which platform selection is guided by the biological question, the lesion feature being tested, and the required balance between control, scalability, and physiological fidelity. We further discuss how modeling oxygen tension, extracellular pH, pressure, mechanics, vascular transport, immune context, and neural interactions can inform niche-directed therapeutic strategies. The central challenge is to build models that reproduce clinically relevant bone lesion states and thereby improve therapeutic prediction, patient stratification, and treatment design.

## 1 Introduction

The bone microenvironment poses a unique challenge for the therapy of bone metastases. Bone metastases therapies frequently underperform because they are primarily tumor-cell-centric, focusing on eradicating malignant cells while largely ignoring the hostile niche that protects them. This mineralized, immunosuppressive microenvironment acts as a sophisticated shield. For instance, the enrichment of extracellular matrix (ECM) components like collagen I/III and fibronectin creates physical barriers that restrict therapeutic penetration (Li et al., 2026; Persson et al., 2026). High tissue stiffness within this niche further reduces the motility and cytotoxicity of infiltrating immune cells, such as T-cells, facilitating immune evasion (Li et al., 2026). Additionally, the bone microenvironment maintains cancer stem-like cells (CSCs), which utilize specialized signaling pathways (e.g., Wnt/β-catenin) to adapt to drug-induced stress and drive therapeutic resistance (Fujiwara and Ozaki, 2016; Menéndez et al., 2021). Physical factors, particularly elevated interstitial fluid pressure (IFP), create a convective barrier that actively opposes the diffusion of chemotherapeutic agents into the lesion core (Gao et al., 2017; Lunt et al., 2008).

Existing *in vitro* and *in vivo* models often fail to capture the full complexity of the clinical lesion, leading to poor translational outcomes. While many systems successfully reproduce the biochemical crosstalk between tumor cells and bone (e.g., the “vicious cycle”), they frequently omit critical physicochemical gradients such as pH and oxygen partial pressure (pO_2_) (Sbirkov et al., 2026; Di Pompo et al., 2021a). For example, regional oxygen tensions in the bone marrow are known to range from <1% to 6%, yet these precise gradients are rarely maintained in standard cultures, which instead rely on static normoxic or uniform hypoxic conditions (Di Giovannantonio et al., 2025). Furthermore, most models do not account for the anisotropic mineral architecture of trabecular bone or the mechanical stimuli derived from interstitial fluid flow and hydrostatic pressure, which are essential for regulating tumor cell mechanotransduction (Persson et al., 2026; Munson and Shieh, 2014). Without integrating these dynamic biophysical cues and the resulting localized acidosis (pH drops), models remain incomplete predictors of how a real lesion will respond to the physiological stresses of treatment (Di Pompo et al., 2021a).

This review does not simply rank preclinical models by complexity. Instead, it argues that the most useful model is the one that reproduces the specific cellular and physicochemical barrier responsible for the biological or therapeutic question under investigation. We aim to explore experimental platforms to test therapeutic strategies for bone metastases and, where relevant, to examine primary bone tumors.

The conceptual focus of this review is the lesion state created by the hostile physicochemical bone niche. We use “hostile niche” to refer to the combined effects of oxygen limitation, extracellular acidosis, mineralized matrix architecture, altered stiffness, pressure, impaired perfusion, and restricted drug transport. These features are discussed not as separate background variables, but as determinants of model selection, therapeutic prediction, and clinical stratification. The review, therefore, links three questions: which hostile niche feature drives the phenotype, which model can reproduce that feature with sufficient control, and which niche-directed strategy is biologically justified.

## 2 What conventional models fail to capture

To understand why therapeutic prediction remains poor, it is necessary first to examine what conventional model systems fail to reproduce. The most important limitations are not only the absence of specific cell types, but also the loss of mineral architecture, oxygen and pH gradients, mechanical constraints, interstitial pressure, and transport barriers that define the metastatic bone lesion.

### 2.1 Limits of 2D monoculture systems

Traditional 2D monocultures on inflexible plastic surfaces do not replicate the sophisticated, mineralized structure of native bone, thereby neglecting critical adhesion signals, such as hydroxyapatite (HA), that influence tumor growth and dormancy. Moreover, these static, homogeneously mixed environments lack the dynamic fluid flow and spatial gradients of oxygen, nutrients, and pH present *in vivo*, which artificially inhibit metabolic reprogramming processes such as glycolysis. The excessive stiffness of planar substrates disrupts mechanotransduction signaling, while the absence of interstitial fluid pressure and dense ECM barriers results in rapid, uniform drug distribution. As a result, these conventional platforms modify native cellular phenotypes, exaggerating therapeutic effectiveness while neglecting to account for penetration-limited resistance mechanisms.

#### 2.1.1 Absence of native three-dimensional (3D) mineralization and its impact on tumor growth

Bone-metastatic breast and prostate cancer cells are often examined on flat plastic (2D) surfaces devoid of hydroxyapatite or mineralized matrix, which inadequately simulates bone. Existing research primarily contrasts these 2D environments with 3D scaffolds, demonstrating that 2D plastic lacks an essential mineral matrix, which significantly alters cellular adhesion. Consequently, these conventional environments artificially exaggerate tumor cell proliferation and fail to reproduce the bone-like mineral signaling required to model authentic cancer-osteoblast interactions and dormant phenotypes (Dozzo et al., 2023; Chen et al., 2026; González Díaz et al., 2022; San Martin et al., 2025; Saraswathibhatla et al., 2023; Mierke, 2024; Huang et al., 2021; Liu et al., 2021; Koedoot et al., 2021).

#### 2.1.2 Uniform culture environments vs. natural nutrient and oxygen gradients

Bone marrow is a densely organized, vascular tissue in which cells experience significant gradients in oxygen, nutrients, and pH, alongside ongoing waste removal. Traditional 2D monocultures on static media subject cells to a predominantly homogeneous, well-mixed environment. This discrepancy affects cell viability, metabolism, and pharmacological responsiveness, diminishing the physiological relevance of investigations centered on the bone marrow. Three-dimensional cultures and organoids inherently establish gradients of nutrients, growth stimulants, oxygen, and cytotoxic chemicals based on cellular positioning, hence more accurately mimicking *in vivo* microenvironments (Suarez-Martinez et al., 2022; Bottaro et al., 2025). In MSC and hMSC aggregates, restricted diffusion of food and oxygen into the core spheroid regions generates hypoxic microenvironments, prompting a transition to glycolysis, a phenomenon not observed in flat 2D cultures under atmospheric oxygen conditions (Habanjar et al., 2021; Rybkowska et al., 2023; Berry et al., 2026). Static culture naturally offers inadequate regulation of metabolic parameters, including the accumulation of by-products such as lactate and ammonia, in contrast to dynamic perfusion methods that continuously refresh the medium (Berry et al., 2026; Huang et al., 2022; Yamada et al., 2023). In spheroids and aggregates, hypoxia and restricted diffusion induce increased glycolytic and mitochondrial activity, altered ROS generation, and variations in stemness markers; these metabolic alterations are partially influenced by nutrition and oxygen diffusion limitations (Habanjar et al., 2021; Berry et al., 2026). Conventional 2D cultures with adequately mixed media under physioxia or normoxia fail to replicate this trend. Primary plasma cells and myeloma cells rapidly perish in conventional 2D monoculture due to the lack of suitable microenvironmental signals and probable nutrient/waste imbalances. 2D cultures uniformly and expose all cells to medications and nutrients, often leading to an overestimation of treatment efficacy and a failure to capture resistance mechanisms associated with hypoxia and gradients reported in 3D models and bone marrow niches (Suarez-Martinez et al., 2022; Bottaro et al., 2025; Gonçalves et al., 2022).

#### 2.1.3 Artificial substrate rigidity and the omission of fluid pressure barriers

In studies of bone metastases, conventional 2D polystyrene plates subject cells to a rigid, planar surface with readily available media, in contrast to the heterogeneous, pressurized, ECM-dense bone-tumor microenvironment. This artificial substrate rigidity profoundly disrupts mechanotransduction signaling, which normally relies on 3D confinement, viscoelasticity, and matrix degradability to govern cellular activity. Moreover, 2D cultures completely omit the fluid pressure barriers inherent to native tumors. *In vivo*, elevated interstitial fluid pressure (IFP) and dense ECM actively compress blood vessels, creating physical obstacles that restrict the transfer of nutrients and chemotherapeutics. The absence of these barriers in well-mixed 2D platforms results in artificially rapid drug distribution to all cells, leading to an overestimation of effective drug exposure and a failure to capture penetration-limited resistance (Saraswathibhatla et al., 2023; Kalli et al., 2023; Jiang et al., 2024a; Feng X. et al., 2024; Micalet et al., 2023; Zhang and Zhang, 2025; Jiang et al., 2022; Hughes et al., 2021).

### 2.2 Limits of standard 3D spheroids and hydrogel systems

Three-dimensional spheroids and hydrogels more accurately replicate tissue architecture and cell–cell/ECM interactions than two-dimensional models; nonetheless, bone metastases develop within a mineralized, perfused microenvironment. Many static 3D systems, devoid of bone mineral signals and regulated interstitial flow, fail to incorporate essential mechanical and transport characteristics that influence tumor behavior in bone. A microribbon-based osteosarcoma model revealed that HA in 3D structures is essential for maintaining in vivo-like signaling and drug resistance; 3D models lacking mineralization or using non-bone-mimicking matrices exhibited diminished physiological relevance (Amos and Choi, 2021). Perfusion bioreactors and bone-on-a-chip technologies are essential for applying controlled shear stress and convective transport, enhancing ECM deposition, and simulating *in vivo* interstitial perfusion, which static 3D cultures cannot replicate (Hughes et al., 2021; Gabetti et al., 2022; Wang et al., 2022).

### 2.3 Animal models and the barriers to clinical translation

#### 2.3.1 Inherent limitations of in vivo platforms in capturing local niche signaling

Animal models replicate numerous emergent characteristics of the tumor microenvironment (TME), such as hypoxia, acidosis, and elevated IFP, yet are ill-equipped to precisely and locally regulate these parameters in bone metastases. *In vivo* cancers, including bone lesions, inherently exhibit acidic and hypoxic areas along with increased IFP resulting from cellular metabolism, aberrant vasculature, and matrix remodeling (Crouigneau et al., 2024; Wang et al., 2024a; Zhang et al., 2025). Analyses of metastasis indicate that limited animal models and the intricacy and expense of advanced mouse strains hinder systematic alteration of microenvironmental settings, hence diminishing confidence that they accurately replicate human metastatic illness (Shi et al., 2024). In the context of bone metastasis, the structure, vascular, hematopoietic, and immunological makeup of mouse bone marrow significantly diverges from that of humans, leading to profound modifications in the bone microenvironment that complicate the precise mapping or regulation of niche-level signals (Kang et al., 2022).

#### 2.3.2 Preclinical mismatches and the underlying drivers of translational failure

A recent study highlights significant non-physiological disparities between conventional preclinical models and the authentic human bone metastasis habitat. The pronounced oxygen gradients observed *in vivo*, approximately 6% “physioxia” in the bone marrow to severe hypoxia in the tumor core, are often disregarded in standard cultures, which typically expose cells to uniform atmospheric oxygen, thereby neglecting hypoxia-induced drug resistance (Pierrevelcin et al., 2022). Moreover, the unique metabolic stressors of native lesions, such as low pH and nutrient deprivation, are virtually unreplicable in 2D systems, where cells lack the physical and metabolic obstacles that often induce the upregulation of multidrug resistance (MDR) proteins (Chiou et al., 2021). The lack of a neoplastic osteoid matrix and abnormal mineralization patterns in numerous 2D and 3D models have prompted researchers to explore bone-mimicking scaffolds, such as β-TCP, to deliver the critical mechanical and biochemical signals that conventional systems cannot provide (Pierrevelcin et al., 2022; Gospos et al., 2025).

Literature on osteosarcoma and breast cancer bone metastases consistently attributes suboptimal clinical translation to “insufficient model systems” that inadequately replicate the authentic bone microenvironment, encompassing its ECM, oxygen gradient, and microarchitectural intricacy (Pierrevelcin et al., 2022; Gospos et al., 2025; Kolahi Azar et al., 2024). In basic 2D models, the lack of hypoxia, acidity, and mineralization barriers results in medications seeming more efficacious, as they are not confronted by the adverse microenvironment that induces MDR, modifies signaling pathways, and limits drug penetration in patients (Chiou et al., 2021; Kolahi Azar et al., 2024). Advanced 3D, bone-mimetic, or humanized models demonstrate that incorporating genuine bone ECM, diverse cell types, and pathophysiological circumstances diminishes perceived therapeutic efficacy, aligning more closely with clinical resistance and highlighting how previously simplistic systems overestimated success (Pierrevelcin et al., 2022; Gospos et al., 2025; Colombo et al., 2021).

## 3 Experimental platforms for modeling the hostile bone niche

### 3.1 Mineralized biomaterial and scaffold systems

To accurately model the physical barriers of the hostile niche specifically, the altered mechanics and dense, mineralized architecture that restrict drug penetration hydroxyapatite (HA) scaffolds, decellularized matrices, and tunable hydrogels serve as essential platforms. Their unique architecture, composition, and stiffness characteristics influence cellular mechanosensation, adhesion, and pharmacological response in bone-mimetic systems. 3D-printed or porous HA scaffolds accurately replicate bone mineral composition and pore structure, thereby facilitating strong adhesion, infiltration, and osteogenic differentiation of stem cells (D’Adamo et al., 2023; Hoseinpour et al., 2024; Yang W. et al., 2024). Rigid, mineral-laden matrices offer substantial mechanical support (compressive moduli frequently within or exceeding the cancellous bone range) and textured, hydrophilic surfaces that promote integrin-mediated adhesion and osteogenic signaling (D’Adamo et al., 2023; Liu X.-L. et al., 2023). Nano-HA and HA composites function as drug carriers, facilitating localized, sustained release of anti-tumor agents and growth factors, significantly influencing tumor cell viability and invasion (Mo et al., 2023; Wang et al., 2024b). Scaffolds derived from decellularized bone ECM or ECM-containing HA composites preserve trace elements and macromolecules that enhance cell adhesion, proliferation, and mineralized matrix deposition compared to synthetic HA alone (Hoseinpour et al., 2024; Cui et al., 2022; Junka et al., 2022). These endogenous proteins and associated factors facilitate physiological integrin and growth-factor signaling, resulting in enhanced in vivo-like mechanotransduction and perhaps more authentic pharmacological responses compared to solely manufactured mineral matrices (Cui et al., 2022; Junka et al., 2022). Hydrogels primarily assess elasticity and viscoelasticity, while the incorporation of nano-HA provides bone-like biochemical and topographical signals; augmented stiffness and mineral content bolster osteogenic signaling, but excessive stiffness may hinder regeneration (Panda et al., 2024). These hydrogels serve as injectable or 3D-printed drug reservoirs, facilitating the controlled release of osteogenic or chemotherapeutic agents, thereby influencing localized drug exposure and sensitivity within a mechanically defined microenvironment (Mo et al., 2023; Pitarresi et al., 2022). Therefore, mineralized scaffolds are most useful when the question depends on matrix composition, stiffness, bone-like adhesion, or local drug retention, but they remain incomplete when vascular flow, immune context, or systemic regulation is central to the phenotype. Mineralized scaffolds should therefore be viewed primarily as matrix-engineering platforms: they are strongest when the experimental question depends on mineral chemistry, stiffness, architecture, adhesion, or local drug retention, but they do not by themselves reproduce patient-specific tissue organization or long-term multicellular self-organization.

### 3.2 3D co-culture systems: modeling cellular crosstalk and phenotypic reprogramming

The hostile bone niche is driven not only by physical forces but by a complex, multicellular ecosystem; when bone-metastatic cancer cells are cultivated in three-dimensional environments alongside osteoblast or osteocyte-lineage cells, they activate specific survival signaling pathways that mimic the protective stromal barriers observed clinically. The direct co-culture of bone-metastatic breast cancer cells with osteo-differentiated human bone marrow stromal cells (osteoblast-like) elevates the receptor activator of nuclear factor-κB ligand/osteoprotegerin RANKL/OPG ratio in cancer cells, a modification not observed with conditioned media or fibroblast co-culture (Arrigoni et al., 2014). This direct interaction also promotes the aggregation of cancer cells without substantially affecting their proliferation or migration, suggesting a contact-dependent osteolytic phenotype rather than mere growth enhancement (Arrigoni et al., 2014).

Osteocytes in three-dimensional spheroids and organ-on-chip environments inhibit cancer cell proliferation and enhance migration through Tumor Necrosis Factor-alpha (TNF-α); metastatic breast and prostate cells counteract this by secreting Transforming Growth Factor-beta (TGF-β), which destroys osteocyte primary cilia (IFT88 pathway) and nullifies the suppressive effect. The ablation of osteocyte TGF-β receptor I reinstates the proliferation-inhibiting and migration-promoting effects of osteocyte signals on cancer cells, illustrating a TGF-β-mediated positive feedback loop that ultimately supports metastatic expansion (Verbruggen et al., 2024). Evidence indicates that the three-dimensional co-culture of estrogen receptor-positive breast cancer cells with osteogenic cells or mesenchymal stem cell-derived osteoblasts results in extensive phenotypic reprogramming. This transition is marked by a substantial change from a luminal to a basal subtype and a considerable decrease in ER signaling, underscoring how the multicellular bone microenvironment fundamentally modifies tumor cell identity and fosters drug resistance. Hybrid EMT, stemness, and signaling via STAT3 and receptor tyrosine kinases (e.g., PDGFRβ). These alterations result in the increase of EZH2/PRC2 epigenomic activity, promoting a more stem-like, ER-independent, therapy-resistant phenotype (Bado et al., 2021).

### 3.3 *Ex vivo* bone explants: balancing structural authenticity with technical limitations

When the experimental goal requires fully intact physical barriers, *ex vivo* bone explant models are highly advantageous because they preserve the complete mineralized matrix and native architecture that dictate interstitial pressure and drug resistance in the hostile niche; nonetheless, they entail compromises in viability, throughput, and variability. Bone explants and organ cultures preserve the native three-dimensional architecture, mineralized ECM, and cell–cell/cell–matrix communication, hence preserving a “natural tumor environment” with osteoblasts, osteoclasts, and marrow cells *in situ* (Kaur et al., 2025). Engineered or human bone structures utilized *ex vivo* are designed to replicate microarchitectural structure, physiological oxygen tension, and intricate cytokine/progenitor combinations, thereby more accurately simulating native bone marrow and tumor–bone interactions than two-dimensional systems (Hughes et al., 2021; Griffin et al., 2023; Bock et al., 2021). Mature bone explants exhibit a restricted lifespan in static culture, encountering challenges in sustaining vitality, particularly in denser or more developed tissue (Decoene et al., 2025). The integration of mechanical loading or dynamic culture can prolong explant viability and stimulate new bone production, however it introduces complexity and necessitates additional equipment (Decoene et al., 2025; Bedell et al., 2023). *Ex vivo* explants, such as human bone discs, generally exhibit low to medium throughput due to the labor-intensive processes of tissue separation, handling, and growth, which span many days to weeks. This differs from higher-throughput manufactured microtissues or printed models, which can be generated more consistently and in greater quantities, but at the expense of complete native architecture (Hughes et al., 2021; Bock et al., 2021). Explant systems inherently exhibit donor heterogeneity in cellular composition, age, and bone quality, complicating standardization and necessitating higher sample sizes for reproducible outcomes (Griffin et al., 2023; Bock et al., 2021; Carvalho et al., 2021). Reviews highlight that explants frequently choose physiologically less pertinent bone (immature, rodent) to minimize variability, hence compromising therapeutic translatability (Decoene et al., 2025).

### 3.4 Bone organoids: replicating patient heterogeneity and facing developmental constraints

Patient-derived bone organoids (PDOs) strive to authentically replicate the interpatient heterogeneity of physicochemical stress such as localized acidic or hypoxic microdomainsmore effectively than conventional 3D cultures; nonetheless, they continue to exhibit immaturity, inadequate vascularization, and challenges in physicochemical control. Bone organoids are constructed from stem or progenitor cells, or cells taken from patients, within three-dimensional scaffolds, thereby emulating the multicellular composition, matrix, and dynamic functions of native bone more accurately than traditional two-dimensional or standard three-dimensional cultures (Hong et al., 2025; Wang et al., 2025; Zhao et al., 2024). PDOs from fibrous dysplasia lesions maintained intralesional molecular and cellular heterogeneity, encompassing fibrosis-associated cell types, transcriptional signatures, and unique genomic/metabolic changes specific to each patient’s lesion (Kim et al., 2024). These PDOs encapsulated essential phenotypic characteristics (e.g., fibro-osseous structure, pro-fibrotic milieu) that are challenging to sustain in more simplistic 3D systems (Kim et al., 2024). Bone tumor organoids derived from patient samples can replicate histopathological features, genetic profiles, and medication responsiveness of the original tumors, hence enhancing customized therapy options compared to conventional models (Hong et al., 2025; Wang et al., 2025).

Consensus evaluations highlight substantial differences in morphology and function between existing bone organoids and human skeletal tissue, indicating insufficient tissue maturity (incomplete bone architecture, remodeling, and functional complexity) (Wang et al., 2025; Zhao et al., 2024). Despite the potential of advanced bone organoids to form blood vessel-like structures, their vascularization remains incomplete and underdeveloped, hence constraining effective nutrition and waste delivery and limiting their size and lifespan (Wang et al., 2025). Organoids frequently lack multisystem collaboration (neural, endocrine, cardiovascular inputs), therefore failing to replicate systemic regulation of bone homeostasis (Zhao et al., 2024). Their development relies on self-organization and fluctuating matrix conditions; shape, size, maturation, and expansion exhibit significant heterogeneity and lack standardization, indicating inadequate regulation of fundamental physicochemical parameters (matrix mechanics, gradients, etc.) (Zhao et al., 2024).

In contrast to scaffold systems, bone organoids are best viewed as self-organizing or patient-informed biological platforms. Their main value lies in preserving interpatient heterogeneity, multicellular phenotypes, and longer-term tissue-like behavior, whereas their current limitations include incomplete mineral maturity, limited vascularization, variable architecture, and less precise control over individual physicochemical variables.

### 3.5 Bone-on-chip and MPS

#### 3.5.1 Fluidic perfusion and spatial compartmentalization

To dynamically model the transport barriers of the hostile niche, bone-on-chip and microphysiological systems (MPS) employ microfluidics and compartmentalization to precisely replicate the impaired perfusion, interstitial pressure, and spatial gradients that drive therapeutic failure. Numerous studies demonstrate that more complex dynamic 3D cultures better mimic dormancy, gradients, and drug responses than simpler static 3D cultures.

Platforms include perfused microchannels to facilitate continuous nutrition and waste exchange while simulating interstitial flow and shear, hence enhancing vascular network development and promoting endothelial activity akin to *in vivo* conditions (Crippa et al., 2022; Zarrer and Taipaleenmäki, 2024). Multi-compartment chips facilitate selective and dynamic paracrine communication among sympathetic neurons, osteoclasts, and bone-tropic breast cancer cells, or between vascular and bone compartments, while inhibiting cell movement through physical barriers and diffusion channels (Zarrer and Taipaleenmäki, 2024; Ji et al., 2023; Pradhan et al., 2023). Certain designs employ leaf vein-like hierarchical flow and endothelialized channels to distribute shear and establish bone-mimetic niches (dormancy versus “vicious cycle”) under regulated perfusion (Seaman et al., 2025).

#### 3.5.2 Multi-lineage cellular incorporation and native topography

Systems incorporate osteoblasts, osteoclasts, mesenchymal stem cells, bone macrophages, endothelial cells, neurons, and tumor cells to establish humanized bone metastasis or bone marrow niches with preserved mineral or mineralized ECM. Bone slices or mineralized bone ECM are included to maintain the natural topographic and physiological signals essential for osteoclast function and cancer-bone interactions (Ji et al., 2023; Pradhan et al., 2023).

#### 3.5.3 Modeling metastatic dormancy and oxygen gradients

A bone-on-chip model featuring three niches (dormancy, perivascular, vicious cycle) used GelMA hydrogels containing MSCs and osteoclasts, along with circulating tumor-conditioned media, to investigate invadopodia formation and the reactivation of dormant lung cancer cells via cortactin signaling. A perfused bone perivascular niche on a chip, created by co-culturing bone marrow stem cells and endothelial cells, promoted sustained slow growth and enhanced treatment resistance in breast cancer cells, mirroring dormancy-like behavior absent in conventional 2D cultures (Zarrer and Taipaleenmäki, 2024). Dynamic, bioinspired co-culture systems, albeit not exclusively microfluidic, were intended for regulated dynamic exposure and demonstrated osteoblast-induced ER + breast cancer dormancy and autophagy, which could be reversed by alterations in the microenvironment, reflecting clinical late relapse (Sánchez-de-Diego et al., 2025).

Reviews highlight that metastasis-on-a-chip and bone-on-chip systems can provide chemical and oxygen gradients, shear stress, and pressure, which standard static 3D models cannot replicate, hence enhancing the prediction of treatment responses (Cocchi et al., 2025). Specialized microfluidic devices can create chronic and intermittent hypoxia gradients, demonstrating that hypoxia enhances cancer aggressiveness, a phenomenon challenging to regulate in bulk 3D cells (Cocchi et al., 2025).

#### 3.5.4 Dynamic mass transport and high-fidelity drug screening

**FIGURE 1:**
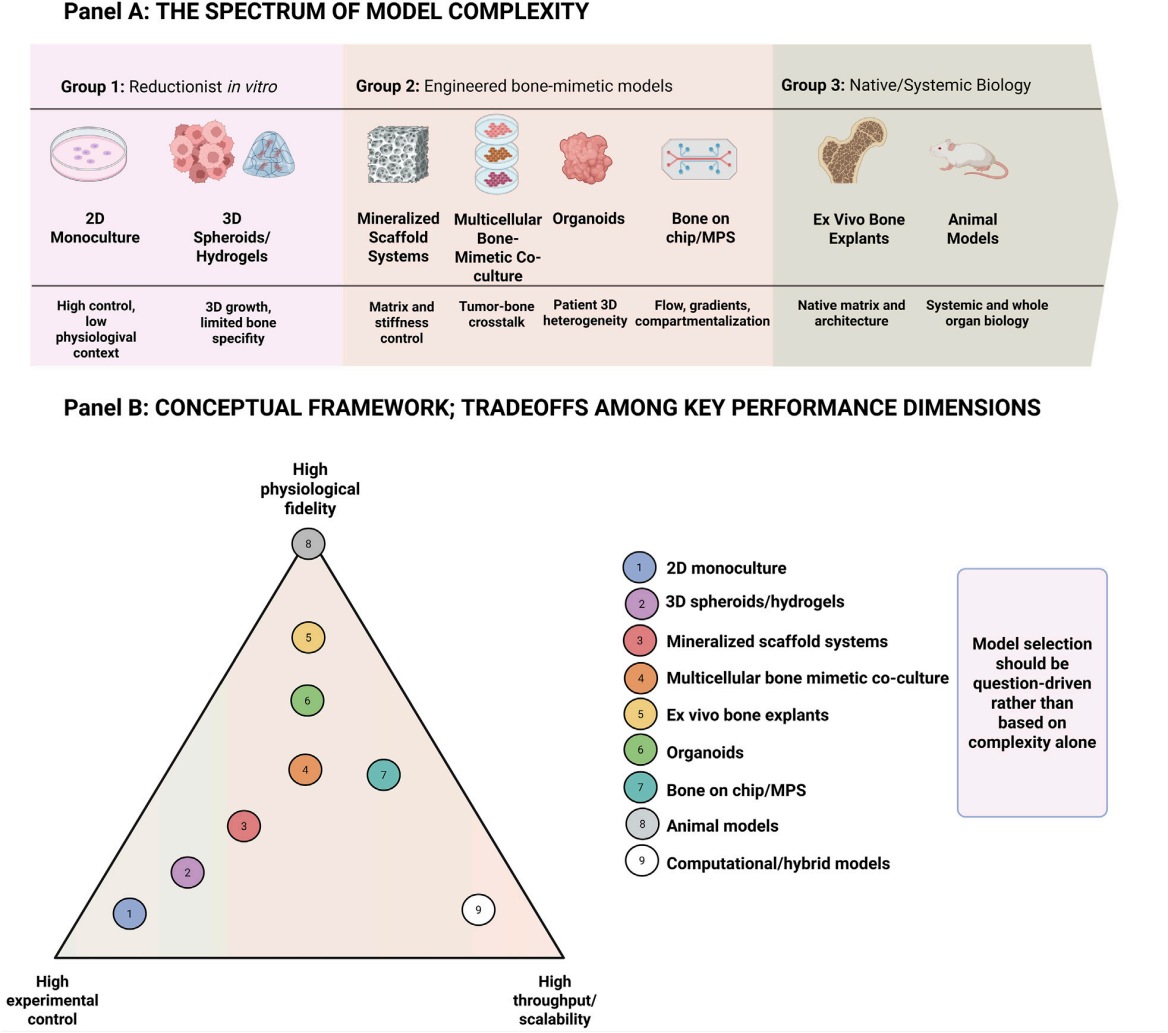
Spectrum of preclinical model complexity and functional tradeoffs in bone metastasis research. Panel **(A)** illustrates the major classes of experimental platforms used to model bone metastasis and bone lesion biology. Reductionist *in vitro* systems, including 2D monoculture and 3D spheroids or hydrogels, provide high experimental control and scalability but reproduce only limited aspects of the bone microenvironment. Engineered bone-mimetic systems, including mineralized scaffolds, multicellular bone-mimetic co-cultures, organoids, and bone-on-chip/microphysiological systems (MPS), progressively incorporate matrix composition, stromal crosstalk, patient-derived heterogeneity, perfusion, compartmentalization, and physicochemical gradients. Native and systemic platforms, including *ex vivo* bone explants and animal models, preserve higher levels of architectural or whole-organ biological complexity but usually provide lower control over individual niche variables. Panel **(B)** presents a conceptual model-selection framework based on three competing performance dimensions: experimental control, throughput/scalability, and physiological fidelity. Simpler systems are strongest for pathway dissection and screening, whereas explants and animal models better preserve native matrix or systemic biology. Bone-on-chip/MPS and computational/hybrid systems occupy intermediate or specialized positions by enabling controlled manipulation of flow, gradients, transport, and parameter prediction. The figure emphasizes that model choice should be driven by the biological question rather than by complexity alone. Created in BioRender. Mohammad, K. (2026) https://BioRender.com/4pynrc6.

Tumor-on-a-chip osteosarcoma models integrate 3D-printed ECM-mimetic matrices with perfusion to enhance the regulation of oxygen and nutrition delivery, while investigating the influence of matrix and flow on morphology, proliferation, and drug metabolism (Zarrer and Taipaleenmäki, 2024; Lu et al., 2024). In a microfluidic bone perivascular niche, fluid flow diminished cancer cell proliferation and decreased the efficacy of the anti-proliferative agent sunitinib relative to static settings, suggesting that flow and vessel-like barriers provide more authentic drug exposure and resistance profiles (Crippa et al., 2022). A perfused osteosarcoma-on-a-chip utilizing 3D-printed dOsECM and a recirculating system was specifically designed to replicate drug responses with greater precision than xenograft or static models, by incorporating bone marrow niches, circulation, and adjustable matrix stiffness (Lu et al., 2024). A micro-osteosarcoma platform featuring concentration-gradient generators facilitated the concurrent evaluation of various chemotherapeutic concentrations under flow, offering scalable and dynamic drug-screening capabilities that exceed those of static 3D spheroids (Colombo et al., 2021). A microfluidic system simulating a vascularized breast cancer-to-bone interface, incorporating self-assembled microvessels, enabled quantification of vascular permeability and neutrophil extravasation, revealing that tumor presence disrupts microvasculature and directly influences drug delivery pathways—characteristics absent in non-perfused three-dimensional constructs (Pradhan et al., 2023). Reviews of metastatic MPS highlight that static models “lack a perfusable network,” which restricts nutrient and waste transport and fails to replicate the diffusion and penetration barriers essential for metastasis and therapeutic response, whereas MPS address these limitations with perfusable microvessels and regulated flows (Zarrer and Taipaleenmäki, 2024; Cocchi et al., 2025; Jackson et al., 2024). In engineered metastatic bone minitissues (lacking complete microfluidics yet more intricate than monocultures), elevated drug dosages were necessary to inhibit cancer proliferation compared to basic spheroid cultures, indicating that enhanced microenvironmental realism diminishes perceived drug sensitivity; microfluidic bone-on-chip models further this by incorporating dynamic flow and compartmentalization (Zarrer and Taipaleenmäki, 2024; Cocchi et al., 2025; Jackson et al., 2024; Cristini et al., 2025). Together, these platforms should be viewed as a continuum of experimental control, physiological fidelity, and scalability rather than as a simple hierarchy of increasing complexity (Figure 1). Because each model captures a different fraction of bone lesion biology, model selection should not be based on complexity alone. A more useful approach is to match the platform to the biological question, the disease stage, and the specific cellular or physicochemical constraint that must be controlled, measured, or therapeutically targeted.

## 4 Fit-for-purpose model selection across the bone metastasis cascade

### 4.1 Defining model fitness for purpose

The novelty of this framework is that it shifts model evaluation from a complexity-based hierarchy to a lesion-feature-based logic, in which each platform is judged by its ability to reproduce the specific hostile niche variable responsible for the biological or therapeutic phenotype.

No single preclinical platform can be considered universally optimal for bone metastasis research, because the features that make a model informative for one question, such as spatial control, multicellular fidelity, or long-term stability, may be the same features that limit its utility for another (Baldassarri et al., 2024). A useful model should therefore be judged not by how many components it contains, but by whether it reproduces the specific biological determinants needed to answer the experimental question with sufficient resolution and relevance (Glaser et al., 2022). This fit-for-purpose view is supported by engineered human bone marrow systems in which selective inclusion of osteoblasts, endothelial cells, mesenchymal stromal cells, hematopoietic stem and progenitor cells, and vascular compartments allowed investigators to identify which niche elements were specifically required to regulate tumor-cell proliferation and hematopoietic remodeling (Persson et al., 2026). In practice, model selection should be driven by the biological process under study, the spatial and temporal scale at which that process unfolds, and the degree to which variables such as flow, oxygenation, stromal composition, and matrix context must be independently controlled or directly measured (Truong et al., 2026). For this reason, the most informative experimental pipeline is often not a single model but a layered strategy in which simpler systems are used for mechanistic dissection, intermediate engineered systems are used for controlled niche interrogation, and more complex platforms are used for validation of emergent phenotypes and treatment response (Sánchez-de-Diego et al., 2025).

#### 4.1.1 Biological fidelity versus experimental control

Biological fidelity and experimental control frequently sit in tension, because models that preserve more native-like multicellular and matrix complexity usually offer less precise control over individual physicochemical variables, whereas microengineered systems can manipulate those variables more tightly but necessarily simplify some aspects of tissue maturity and lesion heterogeneity (Glaser et al., 2022; Truong et al., 2026). This tradeoff is clear in microfluidic bone-on-chip systems, where dynamic perfusion can be engineered within a single device, thereby enabling mechanistic interrogation of cell–cell communication that would be difficult to isolate *in vivo* (Ji et al., 2023). By contrast, more tissue-like biomimetic constructs can recover clinically relevant behavior that is often lost in highly reductionist systems; for example, a 3D printed trabecular-like bone niche supported long-term survival of patient-derived xenograft breast cancer cells, preserved their cycling behavior, and reproduced an in vivo-like drug-response pattern that was not seen under 2D conditions (Han et al., 2021).

#### 4.1.2 Static versus dynamic biological questions

Static or short-window questions, such as early adhesion, initial proliferation, or rapid drug sensitivity comparisons, can often be addressed in simpler 3D formats, especially when those systems permit quantitative imaging and standardized readouts across replicates (Han et al., 2021; Suurmond et al., 2024). However, dynamic biological processes such as dormancy, hematopoietic niche remodeling, vascular trafficking, and stromal microenvironment-mediated resistance require platforms that remain stable over time and permit longitudinal observation of evolving cell states rather than single endpoint measurements (Baldassarri et al., 2024; Glaser et al., 2022; Persson et al., 2026).

#### 4.1.3 Throughput versus physiological realism

**FIGURE 2:**
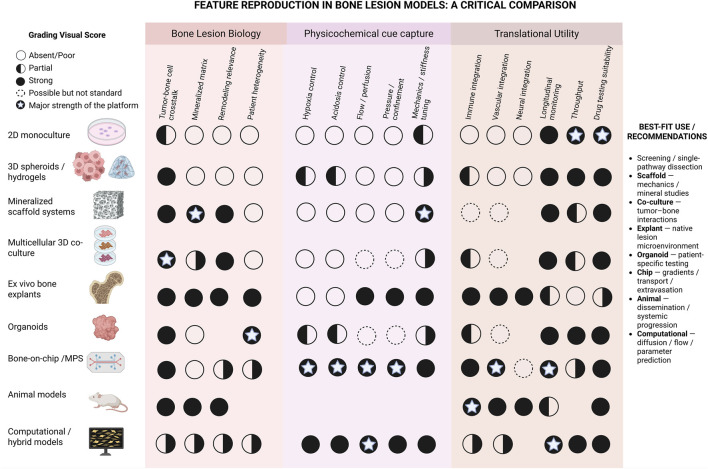
Comparative feature reproduction across bone lesion model platforms. This matrix compares how commonly used bone lesion models reproduce key features relevant to metastatic bone disease, organized into three functional domains: bone lesion biology, physicochemical cue capture, and translational utility. Scoring symbols indicate whether a feature is absent/poorly reproduced, partially reproduced, strongly reproduced, possible but not yet standard, or represents a major strength of the platform. Two-dimensional monocultures offer high throughput and simple longitudinal monitoring but poorly reproduce mineralized matrix, bone remodeling, patient heterogeneity, transport barriers, or multicellular niche interactions. Three-dimensional spheroids and hydrogels improve tumor architecture and drug-response realism but often remain weak in bone-specific mineralization and flow control. Mineralized scaffold systems are particularly strong for matrix chemistry, mineral stiffness, and bone-like mechanical studies, while multicellular co-cultures are better suited for tumor–bone crosstalk and remodeling interactions. *Ex vivo* bone explants preserve native bone architecture and resident cellular complexity but are limited by viability, donor variability, and low throughput. Organoids are valuable for patient-specific biology and heterogeneity but often lack mature vascular, neural, and mechanical organization. Bone-on-chip/MPS platforms are strongest for controlled gradients, perfusion, vascular interfaces, pressure/confinement, and compartmentalized drug testing. Animal models remain essential for systemic dissemination, immune-endocrine context, and whole-organ progression but provide limited control over local pH, oxygen tension, interstitial pressure, and real-time lesion-level measurements. Computational and hybrid models can support transport prediction, parameter testing, and hypothesis generation, but require biological calibration and validation. Created in BioRender. Mohammad, K. (2026) https://BioRender.com/nivi4u2.

Higher-throughput platforms remain valuable because they can expose differences in growth kinetics and drug response rapidly; in a spheroid-based bone metastasis model, for example, multicellular aggregates formed within 24 h, supported confocal quantification over 7 days, and showed both faster cancer-cell expansion and lower cisplatin sensitivity than paired 2D controls (Suurmond et al., 2024). Yet increasing throughput often comes at the cost of physiological realism, since compact spheroid or simplified co-culture systems usually lack bone-specific spatial patterning, perfusable vasculature, and niche compartmentalization, all of which become critical when the goal is to model organ-selective invasion, stromal protection, or lesion-specific drug resistance (Glaser et al., 2022). Conversely, highly complex MPS systems can recover patient-relevant therapeutic behavior but are harder to standardize at scale; notably, a patient-specific prostate cancer bone metastasis MPS incorporating six primary stromal cell types plus an endothelial microvessel reproduced androgen-response sensitivity while also capturing stromal microenvironment-mediated resistance to therapy (Sánchez-de-Diego et al., 2025). These tradeoffs can be made more explicit by comparing each platform according to the niche features it reproduces, the variables it controls, and the translational questions it can realistically address (Figure 2).

### 4.2 Matching experimental platforms to biological stages of bone metastasis

Applying this framework across the major biological stages of bone metastasis reveals that the dominant physicochemical barriers shift as the disease progresses. Early seeding, dormancy, remodeling, therapy resistance, and pain each require different experimental priorities and, therefore, different model systems.

#### 4.2.1 Capturing vascular transit and extravasation

Microfluidic and bone-on-chip systems are particularly suited for studying early metastatic entry because they allow controlled interrogation of tumor–endothelial interactions under defined flow conditions before overt lesion formation (Ji et al., 2023). In a premetastatic bone-on-chip platform, incorporation of vascular-like compartments enabled the analysis of how tumor-conditioned signals modulate interactions between circulating tumor cells and bone-resident cells during early seeding (Ji et al., 2023). Mechanobiological regulation of extravasation can also be resolved in these systems, as demonstrated by a bone-mimetic microfluidic co-culture model in which osteocyte activation via low-magnitude high-frequency vibration reduced breast cancer cell extravasation by approximately 43% (Song et al., 2022). These findings indicate that early metastatic success is not solely tumor-intrinsic but is actively regulated by endothelial barriers and bone-resident mechanosensitive cells (Song et al., 2022).

#### 4.2.2 Modeling early adhesion to mineralized and stromal niches

Once tumor cells enter the bone microenvironment, adhesion to mineralized matrix and stromal compartments becomes a key determinant of colonization success, requiring models that incorporate bone-specific architecture (González Díaz et al., 2023). Spatially patterned 3D systems with engineered bone-like regions allow direct comparison of tumor invasion into bone versus non-bone tissues, thereby isolating bone-selective tropism under controlled conditions (González Díaz et al., 2023). Using such a platform, breast and prostate cancer cells were shown to preferentially invade engineered bone compared to cartilage- or muscle-like regions, while also exhibiting differential remodeling behavior and treatment response (González Díaz et al., 2023). These data suggest that mineralized scaffolds and patterned 3D constructs are most informative for studying early niche engagement after vascular entry has been addressed (González Díaz et al., 2023).

#### 4.2.3 Early adaptation to physicochemical stress

Newly seeded tumor cells must rapidly adapt to local physicochemical conditions, including altered oxygen tension, nutrient gradients, and matrix stiffness, which are not adequately reproduced in conventional static culture systems (Deng et al., 2022). Engineered bone marrow models that incorporate stromal, endothelial, and hematopoietic components provide a platform to examine how these factors collectively regulate early survival and proliferation following seeding (Baldassarri et al., 2024). In such systems, selective inclusion of niche elements has been shown to directly influence tumor-cell expansion and remodeling of the surrounding microenvironment during early colonization (Baldassarri et al., 2024). These findings highlight that early metastatic fate is strongly dependent on the composition and state of the local niche rather than tumor cell properties alone (Baldassarri et al., 2024).

#### 4.2.4 Integrative strategies and systemic validation

Despite advances in engineered systems, *in vivo* models remain essential when the biological question involves organ tropism, whole-body dissemination, and the spatial dynamics of early lesion formation within intact bone. A multimodal mouse study of early breast cancer bone metastasis demonstrated that increased bone remodeling rates correlate with enhanced metastatic seeding and early lesion development, supporting a direct link between host tissue state and colonization efficiency (Young et al., 2024). Such findings underscore the importance of validating *ex vivo* observations within the systemic context, where vascular architecture, immune interactions, and bone turnover cannot yet be fully recapitulated (Young et al., 2024). Taken together, current evidence supports a sequential modeling strategy in which microfluidic systems are used to study vascular entry and extravasation, mineralized 3D systems are used to examine bone-selective invasion and early niche engagement, and *in vivo* models are used to validate homing efficiency and lesion initiation (Jeon et al., 2015). This layered approach allows investigators to disentangle the distinct biological steps of colonization while preserving the ability to test how they interact within the full metastatic cascade (Basu et al., 2025).

### 4.3 Models for dormancy and reactivation

#### 4.3.1 Minimal requirements for modeling dormancy credibly

Dormancy is one of the most difficult states to model in bone metastasis because the relevant phenotype is not simply reduced proliferation, but long-term survival of disseminated tumor cells in a viable, reversible, and niche-dependent quiescent state (Pradhan et al., 2023). A credible dormancy model should preserve tumor-cell viability over extended culture periods while demonstrating sustained proliferative restraint rather than acute toxicity or irreversible collapse (Pradhan et al., 2023). This requirement is illustrated by the 2023 dynamic indirect coculture model of ER-positive breast cancer in which Pradhan and colleagues used bone marrow niche cells to study dormant behavior under prolonged interaction conditions rather than short endpoint assays (Pradhan et al., 2023). Likewise, Chen and colleagues engineered a 3D biomimetic prostate cancer bone niche in which a calcium phosphate scaffold, decellularized ECM, mesenchymal stromal cells, and osteoblasts induced a proliferation-inhibited phenotype marked by G0/G1 arrest and downregulated cell-cycle programs (Chen et al., 2026). These studies suggest that long-term stability, multicellular support, and transcriptomic or phenotypic confirmation of quiescence are more important for dormancy modeling than simple reductions in cell number or Ki-67 labeling alone (Chen et al., 2026).

#### 4.3.2 Endosteal and stromal support of quiescence

Dormancy models are particularly informative when they incorporate osteogenic or endosteal niche elements, because these compartments provide signals that can actively suppress metastatic outgrowth rather than merely failing to support proliferation (Pradhan et al., 2023). In the Pradhan model, human fetal osteoblasts promoted ER-positive breast cancer dormancy and autophagy, whereas mesenchymal stem cells promoted tumor-cell growth, directly showing that different marrow-associated stromal elements drive divergent fates (Pradhan et al., 2023). Nobre and colleagues further demonstrated that periarteriolar NG2+/Nestin + mesenchymal stem cells induce breast cancer disseminated tumor-cell dormancy through TGF-β2 and BMP7 signaling, activating a TGFβRIII/BMPRII–p38-dependent program associated with p27 induction (Nobre et al., 2021). Importantly, genetic depletion of these NG2+/Nestin + mesenchymal stem cells, or conditional loss of TGF-β2 within them, caused awakening and bone metastatic expansion of otherwise dormant p27+/Ki67− disseminated tumor cells (Nobre et al., 2021). Together, these findings indicate that the most useful dormancy platforms are not merely low-growth systems, but niche-resolved systems capable of identifying which stromal compartments actively maintain quiescence.

#### 4.3.3 Modeling reactivation triggers

Reactivation cannot be studied convincingly in static systems alone, because dormant cells often re-enter growth only after a defined perturbation in the surrounding niche (Nobre et al., 2021; Bakir et al., 2025a). One experimentally tractable trigger is bone remodeling: Zhang and colleagues showed that bone metastasis initiation is coupled to bone remodeling through osteogenic differentiation of NG2+ cells, and that pathologic fracture increased metastatic colonization around the injury site (Zhang et al., 2023). These data support the use of models in which remodeling, injury-like cues, or osteogenic state transitions can be dynamically introduced rather than assumed to be constant (Lo et al., 2021). More broadly, the 2025 Nature study by Davenport and colleagues showed that inflammatory perturbation can awaken dormant disseminated breast cancer cells *in vivo*, with influenza and SARS-CoV-2 infections driving loss of a pro-dormancy phenotype and rapid metastatic expansion through an IL-6-dependent mechanism (Chia et al., 2025). Although this study was performed in the lung rather than bone, it strongly argues that reactivation models should be able to accommodate dynamic inflammatory or cytokine-mediated niche disruption rather than focusing only on baseline dormancy maintenance (Chia et al., 2025).

#### 4.3.4 Distinguishing dormancy from slow growth or therapy-induced persistence

An important challenge in this field is that not every low-proliferative population is genuinely dormant, since growth suppression may also reflect stress, nutrient deprivation, or treatment-induced persistence (Chen et al., 2026). For this reason, dormancy models should ideally demonstrate reversible quiescence together with supportive niche dependence. Similarly, the biomimetic prostate cancer bone niche developed by Chen and colleagues was strengthened by a transcriptomic comparison with patient single-cell RNA-sequencing data (Chen et al., 2026). Accordingly, the most convincing dormancy platforms are those that combine long-term viability, niche-specific induction, molecular evidence of quiescence, and an experimentally demonstrable route to reactivation (Nobre et al., 2021).

### 4.4 Models for osteolytic and osteoblastic remodeling

#### 4.4.1 Modeling osteolytic lesions

Models of osteolytic and osteoblastic remodeling must capture tumor–bone reciprocity rather than tumor growth alone, because bone metastases evolve through bidirectional signaling between cancer cells, osteoblast-lineage cells, osteoclasts, stromal cells, and the mineralized matrix (Kumar et al., 2024). Osteolytic models are most informative when they include osteoclast-lineage activity, mineralized substrate, and measurable matrix resorption, because osteolysis is defined by active bone destruction rather than by cancer-cell expansion alone (Kumar et al., 2024). Kumar and colleagues developed a multicellular metastatic bone model that incorporated osteoblasts, osteoclast-related components, 3D matrix stiffness, and mechanical stimulation, allowing osteolysis to be studied as a combined biochemical and mechanobiological process (Kumar et al., 2024). In this model, mechanical compression attenuated breast cancer–induced pro-osteolytic activity, indicating that physical loading can alter the tumor-driven remodeling response rather than merely serve as a passive background condition. These findings support the use of mechanically active 3D systems when the goal is to model osteolytic progression under bone-relevant physical conditions.

#### 4.4.2 Modeling osteoblastic lesions

Osteoblastic disease requires models that can measure abnormal mineral deposition and osteoblast activation, because osteoblastic lesions reflect dysregulated bone formation rather than simple preservation of bone mass (van Santen et al., 2023). In an *in vitro* prostate cancer model, van Santen and colleagues exposed DU145 cells to 2 kPa compression and found that conditioned media from compressed tumor cells decreased osteoclast resorptive activity by 38% while increasing osteoblast mineral nodule formation in four of six donors (van Santen et al., 2023). This study is useful for osteoblastic modeling because it links a bone-relevant mechanical cue to paracrine changes that suppress resorption and enhance mineralization. Therefore, osteoblastic models should include readouts of osteogenic differentiation, mineral deposition, and tumor-derived paracrine signaling rather than relying only on tumor-cell proliferation endpoints.

### 4.5 Models for therapy resistance and drug delivery failure

#### 4.5.1 Intrinsic versus microenvironment-mediated resistance

Therapy resistance in bone metastasis must be modeled as a niche-mediated process, because the hostile physicochemical variables ranging from poor vascular perfusion to dense matrix composition directly alter therapeutic exposure and shield tumor cells from systemic treatments (Sánchez-de-Diego et al., 2025; Dawalibi et al., 2025). This distinction is important because conventional drug screening often measures whether tumor cells are sensitive to a compound, whereas bone-niche models can test whether the drug remains effective when tumor cells are embedded within stromal, endothelial, mineralized, or mechanically constrained microenvironments (Glaser et al., 2022; Han et al., 2021). Models designed for resistance studies should distinguish cell-intrinsic resistance from protection imposed by the surrounding niche, because the same tumor population may show different drug responses depending on whether it is cultured alone or within a bone-like microenvironment (Sánchez-de-Diego et al., 2025). This was demonstrated in a patient-specific prostate cancer bone-metastasis microphysiological system that incorporated primary prostate tumor epithelial spheroids, an endothelial microvessel, and six stromal cell types of the metastatic bone niche (Glaser et al., 2022). In that platform, standard-of-care agents including darolutamide and docetaxel, as well as sacituzumab govitecan, revealed treatment responses that were influenced by the stromal bone microenvironment rather than by tumor epithelial cells alone (Glaser et al., 2022). Notably, sacituzumab govitecan produced greater primary prostate tumor spheroid cell death when stromal cells were absent, indicating that the bone stromal compartment attenuated therapeutic efficacy and directly modeled microenvironment-mediated resistance (Glaser et al., 2022).

#### 4.5.2 Modeling penetration barriers in confined and mineralized lesions

Drug failure in bone metastasis may reflect inadequate penetration into a dense, mineralized, or poorly perfused niche rather than a complete absence of pharmacologic activity against the tumor cell (Han et al., 2021). A 3D bioengineered breast cancer bone-metastasis model generated with a printed trabecular-like scaffold, osteoblast-like conditioning, collagen matrix, and mineralized calcium supported patient-derived xenograft metastatic breast cancer cells and preserved their long-term survival in a bone-like environment (Han et al., 2021). Importantly, patient-derived xenograft cells in this biomimetic bone niche maintained cycling behavior and showed a drug-response pattern similar to that observed *in vivo*, supporting the value of mineralized 3D systems for testing therapy under bone-relevant physical constraints (Han et al., 2021). Spatially patterned 3D bone-metastasis models add another layer of relevance because they can reproduce bone-specific invasion, cancer aggressiveness, cancer-induced remodeling dysregulation, and in vivo-like drug response within the same experimental system (González Díaz et al., 2023). These systems are therefore useful when the therapeutic question involves not only whether a drug kills tumor cells, but whether it remains effective in the spatial context of tumor invasion into bone-like tissue (González Díaz et al., 2023).

#### 4.5.3 Why gradient-aware and flow-aware systems matter

Flow-aware platforms are particularly important for therapy testing because drug exposure, oxygenation, nutrient delivery, and waste clearance are all transport-dependent variables that are poorly represented in static culture. Glaser and colleagues developed a vascularized human bone marrow organ-on-chip containing both endosteal and perivascular niches with dynamic, perfusable vascular networks, allowing niche-specific refactors of doxorubicin and granulocyte-colony-stimulating factor to be evaluated under more physiologic transport conditions. This type of platform is valuable because it permits drug response to be interpreted alongside vascular access, compartmental localization, and niche-specific cell behavior rather than as a uniform exposure across all cells (Glaser et al., 2022).

In bone metastasis specifically, MPS that includes endothelial channels and stromal compartments can help determine whether treatment failure reflects reduced drug access, stromal protection, altered tumor state, or combined effects of all three (Sánchez-de-Diego et al., 2025).

#### 4.5.4 Integrating computational and experimental transport modeling

In bone metastasis research, MPS are essential for distinguishing between true pharmacologic resistance and microenvironment-mediated factors, such as restricted drug access or stromal protection, by integrating perfusable endothelial channels and primary human bone stroma with quantitative readouts of interstitial transport and spatiotemporal cell survival (Glaser et al., 2022; Sánchez-de-Diego et al., 2025). In practical terms, drug-delivery models should incorporate measurable variables such as perfusion rate, diffusion distance, matrix density, endothelial barrier organization, tumor–stroma localization, and spatial patterns of cell death after treatment (Sánchez-de-Diego et al., 2025). High-dimensional approaches can further strengthen these models, as spatially patterned 3D bone-metastasis platforms have already been coupled with single-cell RNA sequencing to identify transcriptional programs associated with bone-specific invasion and therapeutic response (González Díaz et al., 2023). Accordingly, the most informative therapy-resistance models are those that combine bone-specific architecture, stromal and vascular complexity, controlled or measurable transport, and molecular validation of resistant tumor states (Glaser et al., 2022; Sánchez-de-Diego et al., 2025; Han et al., 2021).

### 4.6 Models for pain biology and neuro-bone interactions

#### 4.6.1 Bone pain as a niche-derived biological endpoint

Pain biology in bone metastasis should be considered a direct consequence of niche remodeling rather than a secondary clinical outcome, because tumor-induced changes in bone resorption, acidity, inflammation, and neural architecture can independently drive nociceptive signaling (Bakir et al., 2026a).

Cancer-induced bone pain is strongly associated with osteoclast-mediated bone resorption, which generates an acidic microenvironment that activates acid-sensing ion channels and transient receptor potential (TRP) channels on sensory nerve endings (Lozano-Ondoua et al., 2013). In a murine bone metastasis model, Nagae and colleagues demonstrated that osteoclast-driven acidification was associated with the upregulation of acid-sensing ion channels (ASIC1a and ASIC1b) in sensory neurons, and that pharmacologic inhibition of osteoclast activity with zoledronic acid reduced both the expression of these channels and nociceptive behavior (Nagae et al., 2007). In addition to acidity, inflammatory cytokines released by tumor cells and stromal compartments, including IL-1β, TNF-α, and prostaglandins, have been shown to sensitize nociceptive neurons and amplify pain signaling within the bone microenvironment (Li Z. et al., 2025). These findings indicate that models aimed at capturing bone pain should incorporate osteoclast activity, local pH changes, and inflammatory signaling, rather than focusing solely on tumor burden.

#### 4.6.2 Incorporating sensory and autonomic neurons into experimental systems

Direct tumor–nerve interactions are increasingly recognized as active contributors to metastatic progression, making neural inclusion a critical component of next-generation bone metastasis models. The study demonstrated that sensory nerves promote tumor growth and invasion through neurotrophic signaling, showing that cancer cells can exploit neural pathways to enhance progression in metastatic niches (Wang et al., 2026). Intraosseous tumor models have shown that sensory nerve density increases during metastatic progression, and that tumor-derived factors such as nerve growth factor (NGF) can drive both nerve sprouting and tumor proliferation. Functional studies have further demonstrated that blockade of NGF signaling reduces both nerve fiber density and pain behaviors in bone metastasis models, supporting the role of tumor–nerve crosstalk in both disease progression and symptom generation (Wang et al., 2026). These data support the incorporation of sensory neurons or neurotrophic signaling pathways into experimental systems when the goal is to study tumor progression alongside nociceptive biology (Bakir et al., 2026a).

#### 4.6.3 Linking remodeling, nerve sprouting, and nociceptive signaling

Bone remodeling and nerve remodeling are closely coupled processes, and metastatic lesions can induce aberrant nerve sprouting that contributes to persistent and movement-evoked pain. Bloom and colleagues showed that tumor-induced osteolysis was associated with pathological sprouting of sensory and sympathetic nerve fibers in bone, suggesting that structural changes in innervation accompany and reinforce nociceptive signaling (Bloom et al., 2011). In addition to structural remodeling, tumor-induced release of neurotrophic factors such as NGF, Brain-Derived Neurotrophic Factor (BDNF), and Glial Cell Line-Derived Neurotrophic Factor (GDNF) have been shown to promote neurite outgrowth and increase neuronal excitability in bone metastasis models. Importantly, these neurotrophic signals may be produced not only by tumor cells but also by stromal and inflammatory cells within the metastatic niche, further linking multicellular interactions to pain generation (Lozano-Ondoua et al., 2013; Sun et al., 2026). These findings indicate that models of bone metastasis should incorporate both remodeling-associated structural changes and soluble neurotrophic signaling to capture the full spectrum of tumor-induced nociceptive biology.

#### 4.6.4 Current limitations in neuro-bone modeling

**FIGURE 3:**
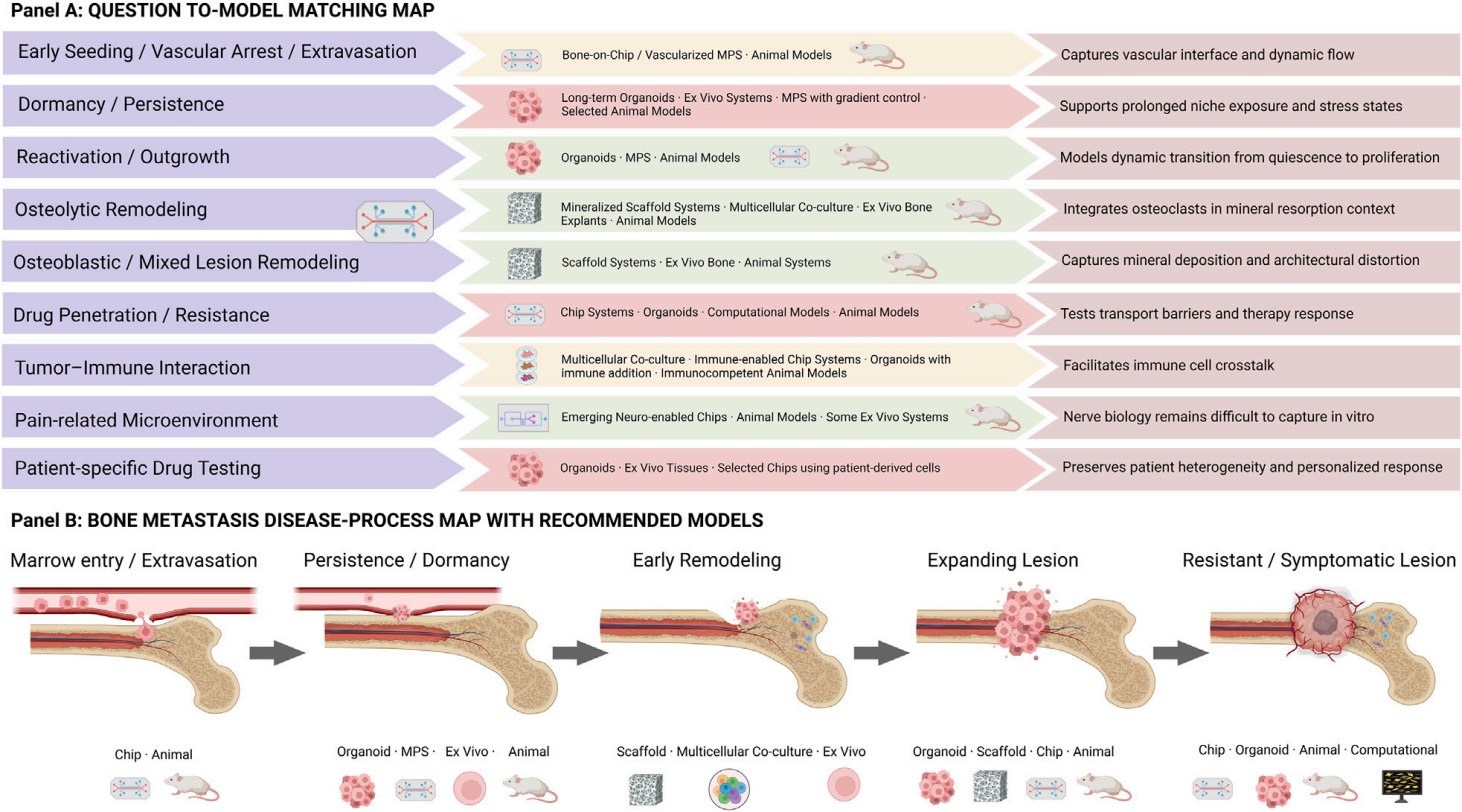
Matching biological questions to appropriate bone metastasis model systems. Panel **(A)** provides a question-driven model-selection map for major biological problems in bone metastasis research. Early seeding, vascular arrest, and extravasation are best addressed using vascularized bone-on-chip/MPS platforms and animal models because these systems capture endothelial interfaces, flow, and tissue entry. Dormancy and persistence require long-term organoids, *ex vivo* systems, MPS platforms with gradient control, or selected animal models that can maintain viable tumor cells under prolonged niche exposure. Reactivation and outgrowth require models that permit dynamic transitions from quiescence to proliferation, including organoids, MPS platforms, and animal systems. Osteolytic remodeling is best modeled using mineralized scaffolds, multicellular co-cultures, *ex vivo* bone explants, and animal models that include osteoclast-mediated resorption. Osteoblastic or mixed lesion remodeling requires mineral deposition and architecture-aware models, especially scaffold systems, *ex vivo* bone, and animal platforms. Drug penetration and resistance studies require systems that reproduce transport barriers, including chip systems, organoids, computational models, and animal models. Tumor–immune interactions are best examined in multicellular co-cultures, immune-enabled chip systems, organoids with immune incorporation, and immunocompetent animal models. Pain-related microenvironmental studies require neural inclusion or systemic validation, because nerve biology remains difficult to reproduce fully *in vitro*. Patient-specific drug testing is best supported by organoids, *ex vivo* tissues, and selected patient-derived chips that preserve interpatient heterogeneity. Panel **(B)** aligns model choice with the temporal evolution of bone metastasis, from marrow entry and extravasation to persistence/dormancy, early remodeling, expanding lesions, and resistant or symptomatic disease. The panel emphasizes that no single model captures the entire metastatic cascade. Instead, a staged modeling pipeline should be used, in which vascularized systems address early entry, organoids and *ex vivo* systems address dormancy and niche persistence, scaffold/co-culture/*ex vivo* systems address remodeling, and chip, organoid, animal, and computational models address resistant or symptomatic lesions. Created in BioRender. Mohammad, K. (2026) https://BioRender.com/x2y6q6w.

Despite increasing recognition of neuro-bone interactions, most *in vitro* bone metastasis models still lack neural components, limiting their ability to study cancer-induced pain or tumor–nerve feedback mechanisms (Yoneda et al., 2021). Reductionist systems using isolated dorsal root ganglion neurons have been useful for studying specific signaling pathways, but they fail to reproduce the combined effects of mineralized matrix, osteoclast activity, stromal signaling, and tumor progression (Leisengang, 2024). Conversely, *in vivo* models provide integrated readouts of pain behavior and central sensitization, but they offer limited control over individual niche variables and make mechanistic dissection more difficult (Curatolo, 2024). Emerging multicellular and microphysiological platforms offer a potential solution by enabling controlled integration of neural, stromal, and bone compartments, although these systems remain technically challenging and are not yet widely standardized (Ashammakhi et al., 2020). Accordingly, future neuro-bone models should aim to combine mineralized scaffolds, osteoclast-driven remodeling, inflammatory signaling, and functional neural readouts such as neurite outgrowth, calcium signaling, or neuropeptide release to more accurately capture both disease progression and symptom biology (Bloom et al., 2011). At present, neuro-bone-tumor modeling should be interpreted as an emerging rather than mature area. Reductionist dorsal root ganglion or sensory neuron co-cultures are technically feasible for studying neurite outgrowth, neuronal excitability, neuropeptide release, or tumor-derived neurotrophic signaling, but they do not recapitulate the full mineralized, remodeling-active bone niche. *In vivo* models remain the most mature platforms for integrated pain behavior and central sensitization, but they provide limited control over local acidity, osteoclast activity, nerve remodeling, and tumor-stromal signaling. Fully integrated nerve-bone-tumor MPS are therefore promising but still exploratory and should be used with careful validation rather than treated as standardized platforms. The question-to-model matching framework is summarized visually in Figure 3 and expanded in Table 1.

**TABLE 1:** Fit-for-purpose model selection for studying biological processes in bone metastasis.

Biological question	Best-suited model type	Key niche components captured	Functional strength/“fitness”	Representative primary studies	Main limitation
Early seeding and extravasation	Bone-on-chip and microfluidic co-culture systems	Flow conditions, endothelial barrier, bone-resident cells (osteocytes, MSCs, osteoclasts), tumor-conditioned signals	Excellent for isolating tumor–endothelial interactions and mechanobiological regulation of extravasation under controlled flow and niche crosstalk	Ji et al. (2023); Song et al. (2022)	Limited systemic context (does not fully recapitulate organ tropism, immune interactions, or whole-body dissemination)
Bone-selective invasion	Spatially patterned 3D bone-like models	Bone-like matrix, mineralized compartments, tissue interfaces (bone vs. cartilage vs. muscle), non-bone comparator regions	Best suited for direct comparison of tumor invasion into bone-like versus non-bone-like tissues, plus linking invasion to bone-specific remodeling and drug response	González Díaz et al. (2023)	Limited ability to model vascular homing, extravasation, and systemic premetastatic niche formation
Dormancy maintenance	Long-term multicellular bone marrow niche models	Osteoblast-lineage cells, mesenchymal stromal cells, indirect niche signaling, quiescence-supportive conditions	Best suited for studying viable, niche-dependent growth restraint and distinguishing dormancy-supportive versus growth-promoting marrow signals	Pradhan et al. (2023); Nobre et al. (2021)	Requires careful validation to distinguish true dormancy from stress-induced slow growth or reduced viability
Reactivation and lesion initiation	Dynamic remodeling or *in vivo* perturbation models	Bone remodeling, osteogenic niche transitions, injury-like cues, stromal state changes	Best suited for testing how changes in the bone microenvironment awaken or support expansion of disseminated tumor cells	Zhang et al. (2023)	Mechanistic isolation is difficult *in vivo*, and *in vitro* perturbations may oversimplify systemic triggers
Osteolytic remodeling	Multicellular 3D tumor–bone models with osteoclast-related readouts	Tumor cells, osteoblast-lineage cells, osteoclast activity, matrix stiffness, mechanical stimulation	Best suited for studying tumor-induced bone resorption as a coupled biochemical and mechanobiological process	Kumar et al. (2024)	Requires validated resorption, matrix, and mechanical readouts rather than tumor growth endpoints alone
Osteoblastic remodeling	Osteogenic/mineralization models incorporating prostate cancer–bone cell signaling	Osteoblast activity, osteoclast resorption, mineral nodule formation, compression-related paracrine signaling	Best suited for studying tumor-induced suppression of resorption and enhancement of bone-forming activity	van Santen et al. (2023)	Findings may be tumor-type and model-specific, especially for prostate cancer–dominant osteoblastic disease
Therapy resistance and drug delivery failure	Vascularized MPS and mineralized 3D bone niche models	Stromal cells, endothelial microvessels, mineralized matrix, perfusion, patient-derived tumor cells	Best suited for separating intrinsic drug resistance from stromal protection, altered exposure, and niche-mediated treatment response	Han et al. (2021); Glaser et al. (2022); Sánchez-de-Diego et al. (2025)	Lower throughput and greater technical complexity may limit standardization and broad screening
Pain biology and neuro-bone interaction	Neuro-bone-tumor co-culture and metastasis-on-chip platforms	Sympathetic or sensory neural components, osteoclasts, tumor cells, inflammatory and paracrine signaling	Best suited for dissecting neural regulation of bone metastatic behavior and linking tumor–bone crosstalk to pain-relevant signaling	Conceição et al. (2022); Park et al. (2024)	Neural integration remains technically underdeveloped and may not capture full behavioral pain responses
Systemic validation of early lesion formation	*In vivo* bone metastasis models	Whole-body dissemination, intact bone remodeling, vascular architecture, immune and endocrine context	Best suited for validating whether mechanisms observed *ex vivo* influence homing, colonization, and early lesion development in an intact organism	Young et al. (2024); Zhang et al. (2023)	Limited control over local oxygen, pH, interstitial flow, pressure, and real-time lesion microenvironment measurements

Table 1 summarizes how different preclinical platforms can be matched to specific biological questions in bone metastasis research. Rather than ranking models by overall complexity, the framework emphasizes functional suitability, key niche variables captured, representative primary studies, and major limitations.

#### 4.6.5 Practical checklist for model selection

A practical way to apply this framework is to begin with the dominant biological question rather than the most complex available model. If the endpoint is drug penetration, the model should include measurable transport constraints, such as diffusion distance, perfusion, matrix density, and spatial drug-response readouts. If the endpoint is immune regulation, the model should include relevant immune subsets and distinguish immune exclusion from direct tumor-cell resistance. If the endpoint is mineral signaling or mechanotransduction, mineralized matrix, tunable stiffness, osteoblast/osteocyte-lineage cells, and mechanical loading should be prioritized. If the endpoint is long-term dormancy or persistence, the platform should support prolonged viability, reversible growth restraint, and niche-dependent reactivation. Thus, model fitness should be judged by whether the platform captures the specific lesion feature being tested, not by the total number of components included.

Once the biological question has been defined, the next step is to determine which features of the hostile bone niche must be engineered into the model. In bone metastasis, the most consequential variables include oxygen tension, extracellular pH, pressure, flow, matrix stiffness, mineral architecture, and multicellular complexity.

## 5 Engineering the physicochemical and multicellular constraints of the bone metastatic niche

After selecting a model system, the next step is to define which features of the hostile niche must be engineered, controlled, or measured. In bone metastasis, fidelity depends not only on cellular complexity, but also on oxygen tension, extracellular pH, fluid flow, pressure, matrix stiffness, mineral architecture, and multicellular organization. These factors shape invasion, dormancy, remodeling, drug penetration, and therapy resistance. Thus, the goal is not maximum complexity but matching each model to the specific niche constraint relevant to the question being studied.

### 5.1 Modeling hypoxia

Hypoxia should be modeled as an active determinant of bone metastasis biology rather than as a generic stress condition, because recent *in vivo* work shows that hypoxia-related activity differs across skeletal metastatic lesions and can associate with lesion morphology and tumor subtype (Das et al., 2024). Hypoxia-related signaling can also alter metastatic organ preference, as Todd and colleagues showed that HIF (hypoxia-inducible factor) signaling in breast tumors controls spontaneous dissemination in a site-specific manner, with HIF pathway alterations producing different effects on bone and lung metastatic burden (Todd et al., 2021).

#### 5.1.1 Oxygen-control platforms

Oxygen-control platforms are valuable because they allow oxygen tension to be treated as an experimentally defined variable rather than an uncontrolled consequence of cell density, medium depth, or diffusion limitation (Zheng et al., 2021). Zheng and colleagues developed an oxygen-concentration-controllable multiorgan microfluidic platform in which dissolved oxygen concentration could be precisely adjusted, demonstrating how engineered systems can expose tumor cells to defined oxygen conditions while preserving organ-to-organ communication (Zheng et al., 2021).

#### 5.1.2 Hypoxic chambers

While conventional hypoxic chambers remain the most prevalent tool for evaluating direct cellular responses and HIF-dependent signaling due to their simplicity, they often fail to separate the effects of low oxygen from confounding factors like nutrient depletion and acidosis (Byrne et al., 2014). Conventional hypoxic chambers may fail to capture the complex oxygen landscape of the bone, as *in vivo* skeletal metastases exhibit significant spatial and subtype-specific heterogeneity in hypoxia activity rather than uniform oxygen deprivation (Das et al., 2024). Therefore, hypoxic chamber experiments are best interpreted as pathway-isolation tools, while more spatially controlled models are needed when the research question involves gradients, regional tumor adaptation, or oxygen-dependent drug penetration (Das et al., 2024).

#### 5.1.3 Oxygen-permeable chip designs and oxygen-gradient systems

Oxygen-permeable microfluidic systems are particularly useful for modeling hypoxia because they can generate controlled oxygen gradients while allowing cells to remain in 3D culture. This type of design is highly relevant to bone metastasis modeling because disseminated tumor cells within marrow or mineralized compartments are unlikely to experience uniform oxygen exposure, especially when they reside at variable distances from perfused vascular structures (Das et al., 2024). Oxygen-gradient chips are therefore better suited than uniform hypoxic chambers for questions involving directional migration, invasion, dormancy localization, or differential drug response across oxygenated and hypoxic microdomains (Ehab et al., 2025).

#### 5.1.4 Measurement strategies

Direct oxygen measurement should accompany hypoxia modeling because nominal incubator oxygen or gas-channel settings may not represent the oxygen concentration experienced by cells inside 3D constructs or microfluidic devices (Bussooa et al., 2022). Bussooa and colleagues demonstrated real-time oxygen monitoring within thermoplastic organ-on-chip devices using integrated optical oxygen sensors, showing that oxygen levels can be followed dynamically rather than inferred indirectly from device settings. On-chip oxygen sensing is especially important in 3D systems because oxygen levels can change with cell density, metabolic rate, perfusion, and diffusion distance, all of which may vary during tumor growth or treatment exposure (Bussooa et al., 2022). In bone metastasis models, oxygen validation should ideally be paired with biological readouts such as HIF activation, hypoxia-marker expression, tumor-cell survival, invasion, or treatment response, because oxygen tension is only meaningful if linked to the phenotype being studied (Todd et al., 2021; Das et al., 2024).

#### 5.1.5 Practical interpretation for bone metastasis models

For pathway-level questions, hypoxic chambers are appropriate because they provide controlled low-oxygen exposure and allow focused testing of hypoxia-responsive signaling (Todd et al., 2021; Das et al., 2024). For spatial questions, oxygen-gradient chips are more appropriate because they can reproduce differential oxygen exposure across a 3D culture region and allow migration or invasion to be analyzed relative to oxygen distribution (Palacio-Castañed et al., 2022). For translational bone-metastasis questions, hypoxia should be integrated with matrix, perfusion, and lesion-subtype context, because *in vivo* skeletal lesions differ in both morphology and hypoxia activity (Das et al., 2024). Thus, the strongest hypoxia models are not necessarily the most complex ones, but those that define oxygen as a controlled or measured variable, match the oxygen design to the biological question, and verify that hypoxia produces a relevant bone-metastatic phenotype (Das et al., 2024; Bussooa et al., 2022).

### 5.2 Modeling acidosis

Acidosis is a defining physicochemical feature of bone metastases because tumor glycolysis, limited perfusion, and osteoclast-mediated bone resorption jointly generate extracellular acidification that influences invasion, remodeling, pain signaling, and therapeutic response (Li T. et al., 2025). Importantly, acidosis in bone is not only tumor-driven but also arises from osteoclast activity, where proton secretion during bone resorption contributes directly to local acidification of the metastatic niche (Yoneda et al., 2011).

#### 5.2.1 Controlled pH media systems

Direct manipulation of extracellular pH using buffered culture media provides a straightforward method to isolate the effects of acidosis on tumor and stromal cell behavior (Kondo and Osawa, 2017). However, global pH adjustment in culture media does not reproduce the spatial gradients and temporal fluctuations present *in vivo*, where cells experience heterogeneous and dynamically changing pH conditions (Korenchan and Flavell, 2019).

#### 5.2.2 Real-time pH sensing and spatial mapping

Real-time, localized pH measurement within 3D tumor models is essential, as extracellular acidity is spatially and temporally heterogeneous and cannot be accurately inferred from bulk media pH values alone (Rizzo et al., 2022). Wang and colleagues developed a fluorescent nanoprobe system capable of mapping extracellular pH gradients within tumor spheroids, demonstrating that acidification increases toward the core of dense tumor regions (Feng Q. et al., 2024). Similarly, microfluidic platforms incorporating pH-sensitive dyes or optical sensors have been used to monitor dynamic changes in extracellular acidity during tumor growth and treatment exposure (Qiu et al., 2024). These approaches are particularly relevant for bone metastasis models, where acidification may vary between osteolytic regions, vascularized areas, and stromal compartments (Di Pompo et al., 2021b).

#### 5.2.3 Modeling metabolism-driven acidosis

More physiologically relevant models allow extracellular acidosis to emerge from tumor metabolism rather than being externally imposed. Previously published research showed that high glycolytic flux and lactate export via monocarboxylate transporters (MCTs) drive extracellular acidification, linking metabolic state directly to pH regulation in cancer cells (Hulikova and Swietach, 2026). In bone metastasis, this mechanism is amplified by limited perfusion and dense matrix conditions, which reduce acid clearance and promote local accumulation of lactate and protons (Qian et al., 2021). Co-culture systems incorporating osteoclasts further enhance physiological relevance, because osteoclast proton pumps actively acidify the resorption lacuna, contributing to tumor-associated acidosis in osteolytic lesions (Di Pompo et al., 2021b). Thus, metabolism-driven models better capture the coupling between tumor metabolism, bone remodeling, and local pH changes.

#### 5.2.4 Distinguishing transient versus sustained acidity

An important consideration in acidosis modeling is the distinction between transient acid exposure and chronic acidic adaptation, as these conditions can produce different cellular responses (Hasegawa et al., 2026). Short-term exposure to low pH may induce stress responses or cell death, whereas prolonged acidosis can select for resistant populations with enhanced invasion, altered metabolism, and increased therapy resistance (Hasegawa et al., 2026). Temporal control of pH has been achieved in microfluidic systems where media exchange or perfusion rate can be adjusted to simulate fluctuating acidity, allowing dynamic exposure paradigms to be tested (Gaebler et al., 2024). This distinction is particularly relevant in bone metastasis, where cycles of osteolysis, perfusion changes, and tumor growth may produce intermittent rather than constant acidification (Lynch and Fischbach, 2014).

#### 5.2.5 Co-modeling hypoxia and acidosis

In bone metastases, hypoxia and acidosis often occur together due to poor perfusion, glycolysis, lactate buildup, and osteoclast activity. Although modeling them separately can clarify mechanisms, combined hypoxia-acidosis systems better reflect endpoints such as metabolic adaptation, invasion, drug resistance, immune suppression, and pain. Microfluidic or perfusion-based models are preferable to static hypoxic chambers because they allow better control of oxygen, pH, metabolite exchange, and real-time monitoring. Ideally, studies should compare normoxic-neutral, hypoxic-neutral, normoxic-acidic, and hypoxic-acidic conditions in mineralized or multicellular bone-like systems.

### 5.3 Modeling pressure and flow

Transport dynamics, including perfusion through the vascular porosity and interstitial fluid flow within the lacunar-canalicular system, are central to bone physiology and thus critically influence the bone metastatic niche by regulating nutrient/oxygen delivery, waste clearance, and mechanosensitive signaling in response to mechanical loading (Cardoso et al., 2013). In bone, where tumor growth occurs within a rigid and spatially confined environment, altered fluid flow and increased solid stress can create heterogeneous microenvironments that influence both tumor progression and therapeutic response (Angeli et al., 2025).

#### 5.3.1 Microfluidic systems for controlled perfusion

Microfluidic platforms provide precise control over fluid flow and shear stress, allowing tumor–endothelial interactions and transport-dependent signaling to be studied under defined conditions (Li et al., 2022). In a bone-mimetic microfluidic system, controlled interstitial flow has been shown to direct tumor-cell migration and influence invasion patterns, demonstrating that flow itself acts as a regulatory cue rather than a passive transport mechanism (Jasuja et al., 2023). These systems are particularly useful for studying early metastatic processes and therapy exposure because they can recreate nonuniform delivery of nutrients, drugs, and signaling molecules across spatially distinct compartments (Li et al., 2022).

#### 5.3.2 Perfusion bioreactors and flow-dependent remodeling

Perfusion bioreactors extend this concept to 3D constructs by enabling continuous medium flow through porous scaffolds, thereby improving nutrient delivery and mimicking aspects of vascular transport. In bone tissue engineering models, perfusion has been shown to enhance cell viability, metabolic activity, and support for osteogenic processes in thick scaffolds (Sailon et al., 2009). For metastasis studies, perfusion systems are particularly valuable when the goal is to evaluate how transport limitations affect tumor growth, stromal interaction, and drug response within a mineralized environment (Jasuja et al., 2023).

#### 5.3.3 Interstitial flow mimetics

Interstitial flow within the bone marrow space is slower and more diffuse than vascular perfusion but can still influence cell migration, signaling gradients, and matrix remodeling (Jasuja et al., 2023). Experimental models that impose low-magnitude interstitial flow have demonstrated that tumor cells can exhibit directional migration along flow gradients, suggesting that convective transport contributes to metastatic dissemination within tissue (Jasuja et al., 2023). Such systems are particularly relevant for studying tumor–stroma interactions and gradient-dependent processes, as they allow control over both flow magnitude and direction (Jasuja et al., 2023).

#### 5.3.4 Compression and confinement systems

Mechanical confinement is a defining feature of bone metastases, where tumor expansion occurs within a rigid mineralized matrix that generates compressive stress and limits tissue deformation (Johnson et al., 2025). Compression-based models have shown that mechanical loading can alter tumor–bone interactions, including modulation of osteolytic signaling and tumor proliferation within multicellular systems (Kumar et al., 2024). In prostate cancer models, mechanical compression has also been shown to influence paracrine signaling between tumor cells and bone cells, shifting the balance between osteolytic and osteoblastic activity (van Santen et al., 2023). These findings indicate that mechanical stress is not only a physical constraint but also a regulator of signaling pathways relevant to metastasis and remodeling.

#### 5.3.5 Integration of flow, pressure, and transport

Flow, interstitial transport, and mechanical stress are tightly coupled in bone metastasis. For example, increased solid stress within tumors compresses blood vessels, reduces perfusion, limits drug delivery, and creates hypoxic and acidic microenvironments that drive aggressive cellular behavior (Johnson et al., 2025). The most informative systems therefore combine controlled perfusion, measurable transport, and mechanical constraints, enabling transport physics to be linked directly to biological outcomes such as invasion, remodeling, and treatment response (Kumar et al., 2024; Manach et al., 2026).

### 5.4 Modeling matrix mechanics and mineralization

Matrix mechanics and mineralization should be treated as regulatory inputs in bone metastasis models, not simply as structural background, because bone-like stiffness, mineral chemistry, spatial architecture, and remodeling capacity collectively shape tumor invasion, osteogenic or osteolytic signaling, and therapy response. González Díaz and colleagues showed this directly in a spatially patterned 3D model where breast and prostate cancer cells preferentially invaded engineered bone-like tissue over cartilage- or muscle-like regions, while the same platform captured bone-specific remodeling dysregulation and in vivo-like drug response (González Díaz et al., 2023).

#### 5.4.1 Tunable stiffness systems

Tunable stiffness systems are useful because they allow investigators to separate the contribution of mechanical resistance from other bone-associated variables such as mineral chemistry, stromal composition, or soluble osteogenic factors. In mechanobiological bone metastasis models, mechanical cues are not passive; Kumar and colleagues demonstrated that mechanical stimulation inhibited breast cancer–induced pro-osteolytic effects, indicating that physical loading can alter tumor–bone signaling and remodeling outcomes (Kumar et al., 2024). Therefore, stiffness-focused models are most appropriate when the biological question involves mechanotransduction, matrix resistance, load-dependent remodeling, or pathways such as integrin signaling, FAK/Src activity, and YAP/TAZ-associated adaptation (Kumar et al., 2024).

#### 5.4.2 Mineralized scaffolds

Mineralized scaffolds are essential when the experimental question depends on bone-specific matrix chemistry, because hydroxyapatite, calcium phosphate, and mineralized collagen provide adhesion and signaling cues that generic hydrogels cannot reproduce. Han and colleagues developed a 3D bioengineered bone metastatic niche using a trabecular-like scaffold, collagen, osteoblast-like conditioning, and mineralized calcium; this platform supported long-term survival of patient-derived metastatic breast cancer cells, preserved their cycling propensity, and reproduced drug-response behavior observed *in vivo* (Han et al., 2021). The value of mineralization is not only structural but also biological, because mineralized matrices can regulate cancer-cell attachment, invasion, dormancy-like persistence, osteogenic signaling, and drug response by changing both the biochemical and mechanical context of tumor growth.

#### 5.4.3 Dynamic remodeling systems

Dynamic remodeling systems are needed because metastatic bone lesions are not static mineral scaffolds; tumor cells stimulate osteoclast and osteoblast activity, and the resulting remodeling changes matrix stiffness, mineral availability, released growth factors, local pH, and physical architecture. A high-fidelity remodeling model should therefore include functional endpoints beyond tumor growth, such as osteoclasts’ resorption, osteoblasts’ mineral deposition, matrix degradation, changes in stiffness, and remodeling-associated secreted factors. This distinction matters because a model may support tumor survival while still failing to reproduce the “vicious cycle” of bone metastasis, where tumor-driven remodeling generates new matrix-derived signals that further support metastatic progression (Kumar et al., 2024).

#### 5.4.4 Architecture-aware design

Architecture-aware design is necessary because bone metastasis is shaped not only by material composition but also by spatial patterning, porosity, tissue interfaces, and the route by which tumor cells encounter and invade mineralized matrix. Accordingly, matrix-focused bone metastasis models should not equate “3D culture” with bone fidelity; the strongest platforms are those that combine tunable mechanics, mineralized chemistry, remodeling-capable cellular components, and spatial architecture matched to the biological question. This integrated approach allows matrix design to be interpreted as a functional variable that regulates invasion, remodeling, and treatment response rather than as a passive scaffold choice (Li T. et al., 2025).

### 5.5 Modeling multicellular complexity

Multicellular complexity is essential in bone metastasis models because tumor behavior is shaped by coordinated interactions among osteoblast-lineage cells, osteoclasts, osteocytes, stromal cells, endothelial cells, macrophages, and neural components rather than by cancer cells alone. Baldassarri et al. developed an engineered human bone marrow model showing that individual stromal niche components regulate breast cancer proliferation and that triple-negative breast cancer cells remodel hematopoietic output within the engineered marrow niche (Baldassarri et al., 2024). A second example comes from a patient-specific prostate cancer bone metastasis microphysiological system incorporating six primary human stromal cell types from the metastatic bone niche, which showed that the stromal microenvironment can alter treatment response and reveal niche-mediated resistance (Sánchez-de-Diego et al., 2025).

#### 5.5.1 Immune inclusion

Immune inclusion is particularly important when the question involves therapy response, inflammatory remodeling, or niche-mediated resistance, because macrophages and osteoclast-lineage cells can regulate both tumor survival and bone turnover. Macrophage-containing models are useful because they allow immune–stromal crosstalk to be studied alongside endothelial, osteogenic, and tumor compartments rather than as an isolated immune assay. This is especially relevant for drug-testing platforms, where stromal and immune cells may alter apparent tumor sensitivity compared with tumor-only cultures (Sánchez-de-Diego et al., 2025). However, immune modeling should remain question-driven, because static addition of immune cells does not necessarily reproduce recruitment, polarization, exhaustion, or myeloid–osteoclast transitions. Therefore, immune inclusion is strongest when paired with functional readouts such as cytokine production, macrophage phenotype, osteoclast differentiation, tumor killing, or treatment modification (Sánchez-de-Diego et al., 2025).

#### 5.5.2 Vascular inclusion

Vascular inclusion is essential for models focused on extravasation, drug delivery, endothelial signaling, and spatial compartmentalization. Glaser et al. developed a vascularized human bone marrow organ-on-chip with endosteal and perivascular niches, showing that perfusable vascular networks enable niche-specific responses to doxorubicin and granulocyte-colony stimulating factor under dynamic transport conditions (Glaser et al., 2022).

Endothelialized platforms are especially useful because they shift the model from a static co-culture to a transport-aware system in which tumor response can be interpreted alongside vascular access, compartment location, and perfusion-dependent exposure. Vascular compartments can also support early-colonization studies, because extravasation and initial marrow entry require tumor interaction with endothelial barriers before direct engagement with bone matrix. In this setting, vascular inclusion should be prioritized when the biological endpoint is entry into the niche rather than later tumor expansion within mineralized tissue (Conceição et al., 2022).

#### 5.5.3 Neural inclusion

Neural inclusion is an emerging priority because tumor cells can interact with sensory and autonomic nerves to regulate both metastatic progression and pain-relevant signaling. Microphysiological platforms also provide a way to isolate neural contributions to bone metastasis. Conceição et al. developed a 3D-printed multicompartment metastasis-on-a-chip system that enabled selective paracrine signaling among sympathetic neurons, bone-tropic breast cancer cells, and osteoclasts, showing that sympathetic neuron and osteoclast signals can increase cancer aggressiveness and inflammatory cytokine production (Conceição et al., 2022). These studies support neural integration when the research question involves neurogenic tumor support, bone pain mechanisms, or tumor–nerve–osteoclast crosstalk. However, neural inclusion remains technically challenging because neurite growth, neuronal activation, neuropeptide release, and pain-like functional readouts require specialized compartmentalized systems rather than simple mixed co-culture (Conceição et al., 2022; Park et al., 2024).

#### 5.5.4 Osteocyte incorporation

Osteocytes should be incorporated when the model aims to capture mechanosensing, load-dependent signaling, or remodeling regulation, because they are the major mechanosensitive cells embedded within mineralized bone (Dabaliz et al., 2026). Song et al. used a microfluidic co-culture platform mimicking the bone–cancer environment and showed that low-magnitude high-frequency vibration activated osteocytes through Piezo1-dependent mechanotransduction and reduced breast cancer extravasation by 43% (Song et al., 2022). Osteocyte–tumor crosstalk can also directly modify metastatic behavior. Sarazin et al. developed a 3D loading model and found that bone-homing metastatic breast cancer cells impair osteocyte mechanoresponse, supporting the idea that tumor cells can disrupt osteocyte mechanobiology rather than simply respond to it (Sarazin et al., 2023). Additional evidence from melanoma bone metastasis shows that tumor-exposed osteocytes can release CXCL5, which enhances melanoma cell migration and invasion through CXCR2; *in vivo*, reducing CXCR2 in melanoma cells or CXCL5 in osteocytes decreased bone metastasis and bone loss (Jia et al., 2024). Together, these studies suggest that osteocyte-containing models are most valuable when the endpoint involves mechanical loading, extravasation, remodeling feedback, or osteocyte-derived chemokine signaling. They also show why osteocytes should not be treated as optional background cells in models claiming to reproduce the mechanically active bone niche.

The same niche features that must be reproduced in preclinical models also represent therapeutic vulnerabilities. Therefore, improved model fidelity is not only a technical goal; it is a route toward identifying which microenvironmental constraints can be targeted to improve drug response, reduce skeletal destruction, and relieve symptoms.

## 6 Therapeutic targeting of the hostile bone niche

### 6.1 Acidity-targeting strategies

**FIGURE 4:**
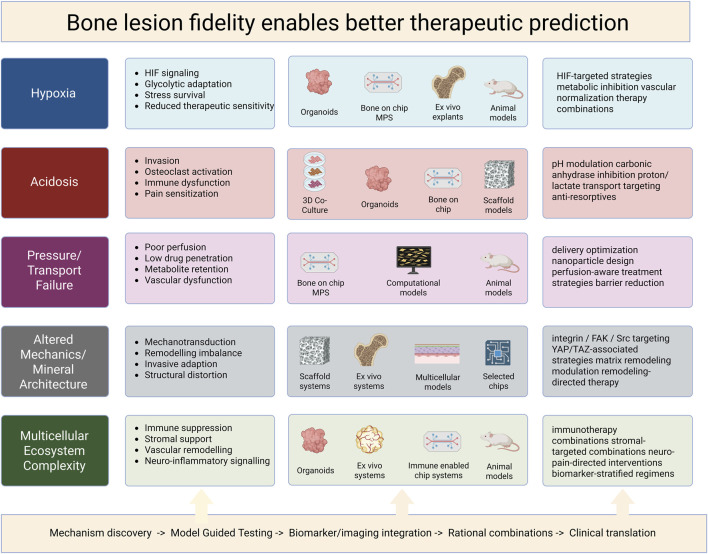
Bone lesion fidelity as a bridge between preclinical modeling and therapeutic prediction. This figure summarizes how reproducing specific hostile features of the bone metastatic niche can improve therapeutic prediction and guide niche-directed treatment strategies. Hypoxia activates HIF signaling, glycolytic adaptation, stress survival programs, and reduced therapeutic sensitivity; organoids, bone-on-chip/MPS platforms, *ex vivo* explants, and animal models are useful for testing HIF-targeted therapy, metabolic inhibition, vascular normalization, and rational combination strategies. Acidosis promotes invasion, osteoclast activation, immune dysfunction, and pain sensitization; 3D co-cultures, organoids, bone-on-chip systems, and scaffold models can support testing of pH modulation, carbonic anhydrase inhibition, proton or lactate transport targeting, and anti-resorptive approaches. Pressure and transport failure contribute to poor perfusion, reduced drug penetration, metabolic retention, and vascular dysfunction; bone-on-chip/MPS, computational models, and animal models are useful for delivery optimization, nanoparticle design, perfusion-aware treatment strategies, and barrier-reduction approaches. Altered mechanics and mineral architecture drive mechanotransduction, remodeling imbalance, invasive adaptation, and structural distortion; scaffold systems, *ex vivo* models, multicellular models, and selected chips can be used to test integrin/FAK/Src signaling, YAP/TAZ-associated pathways, matrix-remodeling modulation, and remodeling-directed therapy. Multicellular ecosystem complexity, including immune suppression, stromal support, vascular remodeling, and neuro-inflammatory signaling, requires organoids, *ex vivo* systems, immune-enabled chips, and animal models to evaluate immunotherapy combinations, stromal-targeted therapy, neuro-pain-directed interventions, and biomarker-stratified regimens. The bottom workflow highlights the translational sequence proposed in the review: mechanism discovery, model-guided testing, biomarker and imaging integration, rational therapeutic combinations, and clinical translation. Created in BioRender. Mohammad, K. (2026) https://BioRender.com/0wk8az5.

A lesion-fidelity framework can help translate preclinical model design into therapeutic prediction. By reproducing the dominant hostile niche feature, hypoxia, acidosis, transport failure, altered mechanics, or multicellular ecosystem complexity, experimental systems can be used to select more rational niche-directed therapeutic strategies (Figure 4).

#### 6.1.1 Carbonic anhydrase inhibition

Carbonic anhydrase IX is primarily located on the extracellular side of the tumor cell membrane. It functions as a catalyst for the CO2 and H2O reaction to produce HCO3- and H^+^, with the protons then exported, thereby acidifying the extracellular space (Lee and Griffiths, 2020). Meanwhile, bicarbonate is reimported into the cell, buffering intracellular pH and providing a favorable environment for the tumor cells. The leading inhibitor used in the trial targeting CA IX is SCL-0111, a sulfonamide, which selectively inhibits CA IX rather than acting broadly (Supuran, 2020). This matters because CA II, expressed in osteoclasts supports Vacuolar-type ATPase (V-ATPase) driven lacunar acidification and resorption, so non-selective inhibitors such as acetazolamide and anti-resorptives such as bisphosphonates act on overlapping pH modulation, raising the prospect of synergy. However, CA II is found in other body cells, so side effects to such treatment can be several, such as tingling, metabolic acidosis, and kidney stones, especially with acetazolamide. For this reason, non-selective CA inhibitors are not used for long-term treatment in cancer patients; instead, the field moves toward emerging CA IX inhibitors (Supuran, 2018).

#### 6.1.2 Proton transport modulation

V-ATPases are the central proton-transport machinery responsible for extracellular acidification in bone metastasis, interacting both within metastatic carcinoma cells that pump H^+^ across their plasma membrane and within tumor-associated osteoclasts whose resorptive activity depends on the same enzyme (Stransky et al., 2016). This dual cellular dependence positions V-ATPase as a uniquely bone-niche-relevant target, particularly the a3 (TCIRG1) isoform, which is required for plasma-membrane localization of the pump in highly metastatic cells and is simultaneously the osteoclast-specific resorption pump (Nishisho et al., 2011). In preclinical bone metastasis models, V-ATPase blockade has consistently reduced skeletal tumor burden: the small-molecule V-ATPase inhibitors FR167356 (a3-selective) and FR202126 each suppress bone metastasis of B16-F10 melanoma and 4T1 breast carcinoma while lentiviral knockdown of the assembly regulator Atp6v1c1 produces broader effects that include reduction of orthotopic and intraosseous tumor growth alongside impairment of mTORC1 signaling (Nishisho et al., 2011; McConnell et al., 2017; Niikura, 2007). V-ATPase–driven acidosis is also a tractable target for cancer-induced bone pain, since intratumoral V-ATPase blockade reduces nociceptive behavior in breast bone-metastasis xenografts via attenuation of stromal NGF/BDNF/IL6/IL8 signaling, and the V-ATPase inhibitor bafilomycin A1, particularly in combination with zoledronic acid, relieves multiple-myeloma bone pain that is otherwise refractory to anti-resorptive monotherapy (Hiasa et al., 2017; Di Pompo et al., 2017). Clinical translation remains constrained by the systemic toxicity of pan-V-ATPase inhibitors such as bafilomycin A1, motivating two parallel strategies: repurposing of high-dose proton pump inhibitors that inhibit V-ATPase activity in tumor cells, and pharmacological development (Fais et al., 2007). NHE1 (SLC9A1) is a major contributor to the alkaline intracellular and acidic extracellular pH of tumor cells, driving invadopodia-guided invasion and metastatic potential, most extensively characterized in breast cancer, with bone-niche-relevant precedent from hexamethylene amiloride’s anti-tumor activity in multiple myeloma and acute myeloid leukemia cells from patient bone marrow and xenografts (Stock and Pedersen, 2017; Jiang et al., 2024b; Yang N. et al., 2024). Clinical translation, however, has been constrained by the cerebrovascular safety profile of cariporide and by recent *in vivo* evidence that prolonged NHE1 inhibition is functionally compensated by upregulation of the Na^+^-HCO_3_^-^ cotransporter NBCn1 (Harguindey et al., 2013; Aaen et al., 2024).

#### 6.1.3 Lactate transport targeting

In bone metastasis, glycolytic tumor cells export lactate and protons through MCT1 and 4, especially leading to microenvironment acidification as well as promoting osteolysis and pain sensation (Singh et al., 2023). MCT 4, specifically, is able to pump these lactate molecules even against high concentrations of extracellular lactate. These allow tumors in hypoxic and dense places to maintain acidity (Sun et al., 2020; Contreras-Baeza et al., 2019). Bone metastasis-prone breast cancer subclones exhibit upregulation of MCT4, GLUT1, and CA9, accompanied by increased lactate efflux and extracellular acidification, suggesting that enhanced glycolytic activity and proton export contribute to bone-specific metastatic adaptation (Di Pompo et al., 2017). In the background of the osteolytic breast cancer model, tumor cells utilize MCT4 to export lactate. Meanwhile, osteoclasts overexpress MCT1 to absorb this lactate, enhancing intracellular acidification, leading to increased collagen resorption and osteolytic activity (Lemma et al., 2017). Therapeutic targeting of in preclinical data demonstrates that inhibition of MCT1 with 7ACC2 in osteoclasts decreases lactate uptake, impairing bone resorption, showing potential as a treatment for bone metastasis (Lemma et al., 2017). Other data exhibits reduced glycolysis, proliferation, circulating tumors cells, and distant metastasis with dual knockout of MCT1/4 as it fully prevents functioning of the lactate transport pathway (Sheng et al., 2023). Pain modulation can also be minimized with MCT4 knockdown in mice demonstrating a pain sensation potential target in bone metastasis (Okui et al., 2023). However, some issues rise as MCT1/4 are expressed in normal tissues and immune cells raising the concern for toxicity (Singh et al., 2023).

#### 6.1.4 Buffering approaches

Systemic buffers include Sodium Bicarbonate, Tris, lysine, Imidazoles heighten buffering capacity where protons are titrated toward physiological pH without affecting the blood pH leading to pH normalization of bone microenvironment. There are also buffer based nanomedicines such as CaCO3, NaHCO3, and liposomes where they also minimize proliferation, migration, modify tumor cells metabolism, and enhance drug uptake (Ibrahim-Hashim et al., 2017; Huang et al., 2016). In the context of bone resorption and cancer-induced pain, these buffering systems inhibit osteoclasts, thereby destroying them and downregulating RANKL (Receptor Activator of Nuclear Factor-κB Ligand) signaling, thereby protecting bone mass (Bailey et al., 2013). Studies have also shown that a reduction in general cancer-associated pain can be achieved with an early bicarbonate trial, suggesting that pH control can modulate nociceptor activation. However, data on bone-specific pain remains limited (Silva et al., 2009; Gillies et al., 2022).

#### 6.1.5 Anti-resorptives as indirect pH modulators

Bone resorption by osteoclasts is mediated by proton secretion via proton ATPases and CIC-7, which lowers pH and triggers cathepsin K release (Nagae et al., 2007). Denosumab and bisphosphonates reduce osteoclastic lysis and resorptive activity by inhibiting the RANK/RANKL pathway, thereby modulating the microenvironmental pH by decreasing proton release (Zhang et al., 2022; Lu et al., 2023; Vaananen, 2005). AS2676293 is a small RANKL inhibitor molecule that suppresses osteoclast differentiation and has been shown to reduce bone metastasis and even metastasis of tumor lines not directly associated with osteoclasts (Nakai et al., 2019). Bone pain caused by cancer cells is due to an acidic environment, in which low pH activates acid-sensing ion channels (ASICs) and TRPV1 neurons. In rat bone metastases models, Zoledronic acid reduced hyperalgesia, spinal c-Fos, and downregulated ASICs receptors in the dorsal root ganglion consistent with bone control of pH and minimized acidotic environment (Nagae et al., 2007; Yoneda et al., 2015).

### 6.2 Hypoxia-targeting strategies

#### 6.2.1 HIF-directed approaches

The bone marrow is constitutively hypoxic, leading to HIF-1α stabilization in resident metastatic carcinoma cells, thereby driving osteolytic progression by suppressing osteoblast differentiation and promoting osteoclastogenesis (Hiraga et al., 2007). Direct HIF-α inhibition has progressed clinically with the HIF-2α–selective inhibitor belzutifan, FDA-approved for von Hippel–Lindau–associated and advanced clear cell renal cell carcinoma, a frequently bone-tropic malignancy and preclinically with HIF-1α modulators such as digoxin, acriflavine, and 2-methoxyestradiol, which suppress lysyl-oxidase–mediated premetastatic niche formation and bone-tropic osteolytic disease in breast cancer xenografts (Das et al., 2024; Choueiri et al., 2021; Wong et al., 2012). HIF activity is also modulated indirectly through HSP90 inhibition, mTOR blockade, or HDAC inhibitors, which restore expression of dormancy-promoting factors such as the leukemia inhibitory factor receptor in hypoxic bone marrow microenvironments. An emerging frontier targets the hypoxia-driven dormancy program of disseminated tumor cells in the bone marrow, where HIF-related transcriptional signatures distinguish quiescent from reawakened metastatic clones (Edwards et al., 2021; Yuan et al., 2024).

#### 6.2.2 Metabolic targeting

The bone marrow’s constitutive hypoxia drives HIF-1α–mediated metabolic reprogramming of metastatic carcinoma cells toward aerobic glycolysis, upregulating glycolytic effectors such as GLUT1, hexokinase 2, lactate dehydrogenase A in bone metastatic breast and prostate cancer. Recent evidence further demonstrates that breast cancer establishes a hyperglycemic bone pre-metastatic niche before colonization, providing a glucose-metabolism vulnerability that is therapeutically exploitable through engineered enzyme nanoplatforms that selectively starve nascent bone metastases (Kwon et al., 2023; Ye et al., 2025; Choe et al., 2015).

Beyond glycolysis, the glutaminase inhibitor telaglenastat (CB-839) is in Phase 1 trials in combination with mTOR blockade, a pairing that addresses the metabolic compensation tumors mount when glycolysis is inhibited, while metformin disrupts RANKL-driven self-renewal of cancer-initiating cells in BRCA1-mutant breast carcinomas with bone-metastasis-initiating capacity, supporting denosumab–metformin combinations. Direct glycolytic-inhibitor monotherapy has been limited by systemic toxicity and tumor metabolic flexibility, motivating combination strategies with HIF blockade, conventional chemotherapy, or radiation (Riess et al., 2021; Cuyàs et al., 2017; Pelicano et al., 2006; Momcilovic et al., 2018; Dabaliz et al., 2025).

### 6.3 Mechanotransduction-targeted approaches

#### 6.3.1 FAK/Src

Focal adhesion kinase and Src are core mechanotransducers that convert matrix adhesion, stiffness, and remodeling cues into survival and resistance signaling in bone metastatic cells. In bone, Fak supports osteoclast-precursor migration and matrix degradation, and Src activity correlates directly with osteolytic metastasis (Győri and Mócsai, 2020; Gunn et al., 2022). FAK-Src and integrin signaling form a positive feedback loop that stiffens the ECM and promotes invasion (Rigiracciolo et al., 2021; Gargalionis et al., 2018). In osteoblasts, Fak inhibition suppresses Akt and osterix, reducing alkaline phosphatases and mineralization and impairing bone formation and skeletal organization (Gunn et al., 2022). In the resistance environment, prostate cancer bone metastasis secretes integrins that activate pFAK-Y397 in nearby tumor cells leading to resistance against cabozantinib through FAK dependence.

Therapeutic targeting of Fak-Src can be achieved by inhibiting Fak Kinase, Src, and Fak in the tumor microenvironment. Experimental agents such as TAE226 in breast cancer bone metastasis and PF-562 in mouse bone metastasis are Fak kinase inhibitors. Oral TAE226 minimizes metastasis effect by affecting osteoclastic cells and RANKL expression. Meanwhile, PF-562 has been shown to impair osteoblastic differentiation and mineralization (Gunn et al., 2022; Kurio et al., 2011).

#### 6.3.2 Integrin signaling

Integrins are adhesion receptors that enable tumor cells to attach to the extracellular matrix. Several integrin subtypes can be altered or overexpressed to support tumor cell proliferation and metastatic colonization. Key ones include a5b1 and avb3 (Kwakwa and Sterling, 2017; Li X. et al., 2025). A5b1 main ligand is fibronectin, and its signaling is responsible for the release of TGF-β and RANKL, favoring bone resorption and magnifying the loop of osteolytic lesions and tumor spread (Li X. et al., 2025). AvB3 main ligands include vitronectin, osteopentin, and osteoclast receptors, and it mainly promotes osteoclast-induced destruction of the bone niche that feeds tumor invasion and expansion. It is very high in bone metastases (Kwakwa and Sterling, 2017). AvB3 antagonism achieved through PSK1404 exhibited direct antimetastatic effects and reduced bone marrow colonization (Zhao et al., 2007). Volociximab is an anti-ITGA5 monoclonal antibody that acts on the a5b1 integrin pathway and has demonstrated reductions in osteolytic lesions, tumor growth, and tumor cell colonization in preclinical models (Pantano et al., 2021).

#### 6.3.3 YAP/TAZ-related strategies

YAP/TAZ are transcriptional co-activators that integrate biological and chemical cues from the bone microenvironment to drive tumor growth, invasion, and therapy resistance. TAZ activation through ABL kinases promotes metastasis by regulating the bone tumor microenvironment. Meanwhile, YAP activation in cancer cells supports metastasis via osteoclastic differentiation. Therapeutic targeting of TAZ includes ABL Kinase inhibition, which reduces bone metastasis by blocking TAZ activation and tumor-bone interactions (Thrash and Pendergast, 2023). Verteporfin, a YAP/TAZ complex inhibitor, has been shown to reduce Ewing sarcoma expansion *in vitro* and lung metastasis *in vivo* (Bierbaumer et al., 2021).

#### 6.3.4 ECM-remodeling enzymes

Matrix Metalloproteinases (MMPs) and cathepsin K are proteolytic enzymes central to bone matrix remodeling. They release TGF-β and other growth factors. MMPs are more central to mobility and enabling tumor colonization of the bone (Piperigkou et al., 2021; Niland et al., 2021). Another MMP, MMP-9, allows for pre-osteoclastic migration and bone resorption. Studies on prostate cancer bone grafts have shown that MMP-9 activity correlates with osteoclastic production. Thus, inhibition of metalloproteases reduces bone destruction and tumor activity (Paolillo and Schinelli, 2019). Broad-spectrum MMP inhibitors were preclinically effective in modulating tumor activity and bone degradation. However, they failed in clinical trials due to poor selectivity and side effects (Prakash and Shaked, 2024; Kang et al., 2022). Newer strategies focus on selective targeting of MMPs (2,9,14) and delivery to the bone tumor niche (Prakash and Shaked, 2024; Kang et al., 2022).

### 6.4 Improving penetration and delivery

Key barriers in the bone microenvironment include blood-bone marrow barriers, impaired bone remodeling, low perfusion, and an acidic bone niche due to the tumor. Nanoparticles sized 70–100 nm can be transported to the bone marrow via sinusoidal capillaries by exploiting the leaky-vessel structure of the bone metastasis niche (Bakir et al., 2025b). Bisphosphonates can act through active targeting strategies, in which they have a high affinity for the mineral matrix of bone, specifically hydroxyapatite. There is also the Metal-Organic Frameworks technique, in which an MOF binds bone via its targeting shell, and MMP-sensitive peptide links allow the carrier to detach and penetrate deeper into the tumor. An example of this is detachable D8-peptide/HA-ZIF-8 carriers, which showed acid/MMP drug release, allowing it to favor acidic bone metastasis niche (Yang et al., 2022).

Because these niche abnormalities rarely act in isolation, therapeutic targeting of a single pathway is unlikely to be sufficient for many patients. Rational combination strategies should therefore link a standard anticancer therapy to a defined microenvironmental barrier and then measure whether that barrier has been modified.

## 7 Rational combination strategies

Niche-directed therapy should not be treated as a separate line of care, as its value lies in changing the lesion state that blocks standard treatment. What lesion state is blocking treatment? Which niche variable can be changed with a measurable clinical gain? These two questions should guide the design of the combination. Bone metastases progress through vascular entry, marrow survival, dormancy, remodeling, overt growth, and pain, so combinations should be selected based on the dominant lesion biology rather than on drug class alone. Recent reviews define bone metastasis as an evolving tumor-niche process, in which osteoclasts, osteoblast-lineage cells, matrix, vascular niches, hypoxia, acidity, and immune exclusion create different resistance states within the same patient (Kang et al., 2022; Satcher and Zhang, 2022). A rational combination links one standard anticancer treatment to one defined niche barrier, then measures whether that barrier changed. This avoids indiscriminate multi-drug escalation and keeps the study focused on a testable mechanism, a lesion phenotype, and a patient-relevant endpoint.

### 7.1 Anti-resorptives plus physicochemical modulation

Bisphosphonates and denosumab remain the clinical anchor for skeletal protection, but their main action is control of osteoclast-driven bone loss rather than full reversal of the acidic, hypoxic, mechanically abnormal lesion state (Lu et al., 2023). Denosumab or zoledronic acid can reduce skeletal events in metastatic breast cancer, and de-escalation data show that dose scheduling can be adapted after initial disease control in selected patients (Aaen et al., 2024; Singh et al., 2023). A rational next step is to pair anti-resorptives with agents that reduce proton production, lactate export, carbonic anhydrase activity, or matrix-dependent signaling. This pairing has a clear biological logic, whereby osteoclasts release protons during resorption, tumor cells add lactate and carbonic acid, and the combined acid load supports invasion, pain, immune suppression, and drug failure (Das et al., 2024; Yuan et al., 2024; Peppicelli et al., 2025). Anti-resorptives can reduce the osteoclasts’ contribution to this acid burden, but they do not directly correct tumor glycolysis, hypoxia-driven pH regulation, or matrix stiffness. The most coherent combination is anti-resorptive therapy plus acidity or mechanotransduction targeting in patients with active osteolysis, high bone turnover markers, lytic imaging patterns, and pain linked to resorption. Along with that, trial readouts should include parameters like skeletal events, bone turnover markers, pain scores, analgesic use, lesion pH or hypoxia markers where available, and lesion-level imaging response. Bone-targeted nanocarriers can further refine this strategy by increasing drug residence near hydroxyapatite and metastatic bone surfaces, rather than exposing the whole body to a pH or mechanics inhibitor (Bakir et al., 2025b; Wu et al., 2024).

### 7.2 Standard therapy plus microenvironment normalization

Standard systemic therapy should be tested alongside niche modulation in lesion states where the microenvironment impedes exposure or response. Chemotherapy fails in bone, in part, due to poor perfusion, high interstitial pressure, stromal protection, and acidic trapping of weakly basic drugs. Endocrine therapy and targeted therapy also support a protective marrow niche that can keep disseminated cells alive during treatment (Satcher and Zhang, 2022). Radiotherapy is weakened by hypoxia, and HIF-driven programs can shift tumor metabolism, angiogenesis, osteolysis, and immune tone (Das et al., 2024; Yuan et al., 2024). Normalization strategies require sequence and purpose: a short priming phase with vascular modulation, acidity modulation, hypoxia, or matrix modulation can make a lesion more penetrable before chemotherapy, endocrine therapy, targeted therapy, or radiotherapy act on tumor cells under better transport conditions. Similarly, the order of treatment matters, as a transport or oxygenation intervention given too late only documents failure, whereas a brief pre-treatment window can test whether the lesion becomes more drug-accessible or radiosensitive. Nanomedicine offers a practical route, especially for drugs that benefit from mineral targeting, pH-sensitive release, or protease-sensitive detachment within the metastatic niche (Bakir et al., 2025b; Wu et al., 2024). The same logic applies to radiotherapy, where hypoxia imaging and oxygen-related biomarkers can select patients for dose adaptation, radiosensitization, or HIF-directed combinations (Yuan et al., 2024; Alenezi et al., 2025; Costin and Marcu, 2024).

### 7.3 Immunotherapy combinations

Bone metastases are often treated as an anatomic site, but they also represent an immune context. The marrow niche contains myeloid cells, osteoclast-lineage cells, TGF-beta-rich matrix stores, hypoxia, acidity, and dense stromal barriers that can reduce T-cell entry and function (Joseph et al., 2023). Clinical data in non-small-cell lung cancer (NSCLC) show poorer outcomes for patients with bone metastases treated with immune checkpoint inhibitors, and mechanistic reviews describe bone metastases as a setting of immune resistance rather than simple tumor burden (Joseph et al., 2023; Zhu et al., 2024; Zhao et al., 2025). This supports combination trials that pair immune checkpoint blockade with bone-targeted therapy, anti-angiogenic therapy, or niche-directed modulation (Bakir et al., 2026b). In NSCLC cohorts, immune checkpoint inhibitors given with bone-targeted therapy or anti-angiogenic agents have shown signals that support this direction, and treatment of bone lesions with pembrolizumab-based regimens has produced lesion-level responses in selected cases (Bongiovanni et al., 2021; Xie et al., 2023; Asano et al., 2022). Biomarker work in NSCLC with bone metastases also suggests that inflammatory and nutritional indices can track response risk during immune checkpoint inhibitors (ICI) therapy, which supports serial immune-state monitoring rather than baseline PD-L1 alone (Asano et al., 2022). The key principle is not to add immunotherapy to every bone-metastatic case. It is to identify lesions in which hypoxia, acidity, myeloid exclusion, or active osteoclast signaling make immune escape a niche problem. In those lesions, the niche intervention should be selected to open immune access, reduce suppressive myeloid activity, or lower metabolic stress before response is judged.

### 7.4 Disease-stage-specific interventions

Combination therapy choice should follow disease stage. At the outset, early colonization requires strategies that disrupt homing, endothelial adhesion, marrow retention, and pre-metastatic remodeling. In contrast, a state of dormancy requires either preservation of a growth-restraining niche or eradication of dormant cells with minimal damage to the marrow reserve. As the disease advances to overt lesions, treatment must pivot to combined control of tumor burden, remodeling, pain, and transport failure. For symptomatic palliative disease, the final goal is relief of pain, prevention of fracture, and control of lesion progression, which is best achieved by evaluating radiotherapy, anti-resorptives, procedures, and niche-directed analgesic mechanisms together. A stage-specific design avoids the common error of testing a late osteolysis drug against an early dormancy endpoint, or testing an immune drug in a lesion where drug entry, acidity, or marrow suppression has not been addressed. Under this framework, early trials utilize short biologic endpoints. Dormant disease trials employ long observation and sensitive residual disease measures, and palliative trials focus on pain control and function as primary outcomes. While clinical trial reviews in prostate cancer and mixed bone-metastasis populations show that current trials remain too heterogeneous in intervention types and endpoint choices, transitioning to stage-matched designs offers a clear path to connect mechanism, biomarker, and patient benefit (Zhou et al., 2024; Shen et al., 2023). For niche-directed therapy to become clinically useful, the dominant lesion state must be measurable in patients. Biomarkers and imaging are therefore essential for identifying which lesions are hypoxic, acidic, poorly perfused, actively remodeling, immune-excluded, or mechanically unstable, and for determining whether treatment has changed these states.

## 8 Biomarkers, imaging, and clinical translation

Biomarkers for niche-directed therapy must describe the lesion, not only the cancer lineage. A bone metastasis carries a tumor genotype and measurable niche states, including hypoxia, acidity, osteolysis, osteoblastic repair, matrix remodeling, neural sensitization, immune exclusion, and poor transport. Clinical translation depends on linking these states to treatment selection and to early pharmacologic change. A useful biomarker identifies whether the lesion is hypoxic enough to justify hypoxia targeting, whether osteoclast activity is driving pain and acid load, whether poor perfusion is blocking drug entry, whether the immune compartment is excluded or exhausted, and whether a bone-targeted carrier reaches the active interface. These measures should also be repeatable over time, because the same lesion can shift from osteolytic expansion to sclerotic repair after treatment. Rather than mere description, the true goal of a biomarker is patient selection, early proof of target engagement, and the prediction of benefit.

### 8.1 Imaging the hostile niche

Imaging is central because bone lesions are spatially mixed and biopsy samples capture only a small region. Hypoxia imaging with nitroimidazole PET (positron emission tomography) tracers, oxygen-sensitive MRI approaches, and radiomic surrogates can help identify lesions in which oxygen limitation is likely to alter radiotherapy, immunotherapy, or drug metabolism (Alenezi et al., 2025; Huang et al., 2025). Because these methods are not interchangeable, each must be leveraged for its specific strengths: PET provides a molecular readout of hypoxic cell burden; MRI (magnetic resonance imaging) delineates structure, perfusion, marrow replacement, and diffusion; and radiomics extracts spatial patterns from routine images. Metabolic imaging further refines this picture by uncovering highly specific niche characteristics. For instance, while Fluorodeoxyglucose reflects glucose use and inflammation, NaF highlights active osteoblastic turnover and mineral exchange. Similarly, Prostate-Specific Membrane Antigen or choline imaging can map prostate cancer bone disease, and when deployed via hybrid PET/MRI, can combine these functional tumor readouts with exquisite marrow and soft tissue detail (Zamani-Siahkali et al., 2024; Ulusoy et al., 2024; Ouvrard et al., 2024). A comparative whole-body PET/MRI study found that fused contrast-enhanced T1-weighted PET/MRI had high diagnostic performance for bone metastasis detection, supporting combined structural and metabolic imaging for lesion mapping (Ulusoy et al., 2024). For niche-directed trials, imaging must move beyond mere detection to actively record baseline niche states, confirm target engagement, and differentiate flare or repair from true progression. Aligning imaging schedules with the underlying biology of the intervention is critical to this approach; while changes in perfusion or hypoxia can be assessed early, capturing the repair of mineralized bone and reductions in skeletal events naturally requires a longer follow-up window.

### 8.2 Biomarker opportunities

Acidity-related markers include carbonic anhydrase IX, monocarboxylate transporters, lactate dehydrogenase programs, extracellular vesicle patterns linked to acidic stress, and future pH-sensitive imaging probes (Stock and Pedersen, 2017; Vaananen, 2005). Similarly, hypoxia-associated markers encompass HIF-regulated signatures, Vascular Endothelial Growth Factor (VEGF)-linked vascular patterns, hypoxia PET uptake, and radiomic phenotypes that reflect oxygen limitation (Yuan et al., 2024; Alenezi et al., 2025; Costin and Marcu, 2024; Huang et al., 2025). Beyond these microenvironmental factors, ECM and mechanics markers include collagen organization, Lysyl Oxidase (LOX) activity, integrins, FAK-Src signaling, YAP/TAZ activation, and matrix-derived fragments, while remodeling-linked markers track CTX, Procollagen Type 1 N-terminal Propeptide (P1NP), bone alkaline phosphatase, Tartrate-Resistant Acid Phosphatase 5b (TRACP5b), RANKL-OPG balance, and imaging of lytic or sclerotic turnover (Lu et al., 2023; Elshimy et al., 2025). Crucially, none of these markers should be interpreted in isolation, as their biological relevance depends entirely on the structural context. For instance, a high CTX level carries a vastly different meaning in a lytic, painful, acidic lesion than in a treated sclerotic lesion showing signs of repair. Likewise, a hypoxic PET signal must be contextualized differently in a poorly perfused lesion than in one that is metabolically active yet highly vascular. Ultimately, an effective biomarker panel must combine tumor, bone, vascular, immune, and symptom measures, while strictly separating prognostic markers from predictive ones. After all, a marker that merely identifies a poor outcome is insufficient for niche-directed therapy; the same marker must identify a dynamic lesion state that actively changes in response to the selected intervention.

### 8.3 Stratifying patients for niche-directed therapy

Patient selection should be both lesion- and stage-specific, meaning that therapeutic priorities must shift based on the dominant pathology. Patients with high-turnover osteolytic lesions are the clearest candidates for anti-resorptive plus acidity modulation, whereas those with hypoxic lesions warrant HIF-directed therapy, radiosensitization, or altered radiation planning (Das et al., 2024; Yuan et al., 2024; Elshimy et al., 2025). When facing immune-cold bone disease, high inflammatory indices, or poor ICI response patterns, the approach must pivot toward immune combinations that target the niche barrier (Joseph et al., 2023; Zhu et al., 2024; Asano et al., 2023). Furthermore, diffuse marrow disease requires protecting the hematopoietic reserve and optimizing drug delivery, rather than relying on aggressive local escalation alone, whereas oligometastatic disease opens the door to localized treatment combined with systemic niche modulation. However, effective stratification must also account for the profound discordance between distinct lesions. A single patient can easily present with a hypoxic lytic lesion, a sclerotic healing lesion, and a marrow-dominant lesion; consequently, a treatment strategy derived from a single scan label is insufficient. Instead, a practical stratification system must classify the dominant lesion state, document the most symptomatic lesion, and mark those at high risk for fracture or cord compression. This approach yields a comprehensive treatment map rather than a single patient label.

### 8.4 Clinical-trial design considerations

Trials of niche-directed therapies must utilize endpoints that align precisely with their intended biological mechanism rather than relying solely on traditional RECIST criteria. For example, an agent mitigating acid-mediated pain should be evaluated using pain scores, analgesic use, and osteoclast activity, whereas a vascular-normalizing drug should be evaluated by perfusion, local drug exposure, and shifts in hypoxia. Similarly, remodeling therapies should focus on skeletal events and the structural quality of bone repair. Lastly, radiotherapy combinations should prioritize localized lesion management and pain control, an endpoint that real-world data confirms is both highly quantifiable and clinically meaningful in large bone-metastasis cohorts (Hovenier et al., 2026).

To operationalize these endpoints, biomarker-enriched trials can use adaptive designs, lesion-level imaging, serial liquid biopsies, and paired tissue sampling in accessible lesions. The most robust clinical trials will explicitly define the baseline niche abnormality at entry, demonstrate early target engagement, and subsequently test whether these microenvironmental shifts translate into improved tumor control, skeletal protection, or patient quality of life. This structured framework moves niche-directed therapy into the realm of rigorous, testable science, rather than leaving it as a vague add-on to standard care. Finally, this specialized focus requires a distinct approach to data reporting. Trial data should present lesion-level outcomes alongside broader patient-level metrics, given that the individual treated bone lesion is the exact unit in which niche modulation is most evident. Safety reporting must be similarly bone-specific, mandating close monitoring of marrow reserve, hypocalcemia, renal function, osteonecrosis risk, and fracture-healing integrity.

These translational requirements point toward a broader research agenda. The next-generation of bone metastasis research must integrate engineered model systems, lesion-level biomarkers, patient-derived materials, and clinically meaningful endpoints into a more predictive framework for therapy development.

Bringing niche-directed therapies into clinical practice remains challenging. Many proposed targets, including metabolic regulators, matrix enzymes, and mechanotransducers, are also important in normal tissues, raising concerns about systemic toxicity. Drug delivery is another major barrier because bone metastases differ widely in vascularity, stromal composition, and mineral density. Although bone-targeted delivery may improve local drug exposure, it could also disrupt normal bone remodeling or hematopoiesis. For these therapies to succeed, patients will need to be selected based on lesion-specific biomarkers, with trials designed to confirm target engagement within the metastatic niche rather than relying only on systemic drug levels or overall tumor burden.

## 9 Roadmap for next-generation research

Future experimental models must transition from static, simplified systems to dynamic platforms that recreate the physicochemical complexity of the bone marrow. These next-generation systems should feature tunable oxygen levels to mimic the native hypoxic marrow environment and tunable pH to simulate the acidosis common in malignant lesions (Di Pompo et al., 2021a; Di Giovannantonio et al., 2025; McKeown, 2014). Furthermore, integrating interstitial fluid flow and hydrostatic pressure within a mineralized matrix is essential to capture the mechanotransduction signals that drive tumor progression (Di Carlo, 2026). Crucially, these models must evolve beyond tumor-centricity to incorporate multicellular complexity, including immune, vascular, and even neural components, while providing longitudinal readouts through real-time biosensors to track therapeutic responses over time (Mohammadzadeh et al., 2026; Bakir et al., 2026a).

To ensure these advanced models translate to clinical utility, the field must prioritize standardization. Current research often suffers from a lack of reproducibility due to variations in scaffold materials, cell densities, and medium compositions across laboratories (Liu et al., 2025). Future reporting standards should mandate the precise documentation of physiological parameters, including pH, oxygen tension, matrix stiffness, mineral content, and flow rates. Establishing unified benchmarks for model fidelity and reproducibility will allow for the integration of data across different studies, fostering the reliable comparison of novel drug candidates and enabling the construction of highly biomimetic “bone-on-chip” platforms for high-throughput screening (Di Carlo, 2026; Liu et al., 2025).

Bridging the gap between engineering and biology will require forming integrated teams that combine expertise in oncology, materials science, and computational modeling. A central pillar of this effort should be the utilization of patient-derived materials, such as patient-derived organoids (PDOs) and explants, which better reflect the intratumor heterogeneity and molecular landscapes of clinical cases (Liu Y. et al., 2023). By combining high-resolution multi-omics with functional phenotyping—where the real-time behavior of live patient cells is tested against various drug regimens—researchers can achieve a more predictive “functional precision medicine” framework that identifies effective therapies even in the absence of actionable genomic mutations (Zhang et al., 2026).

Ultimately, the next-generation of successful therapies for bone metastases will likely depend on a fundamental paradigm shift: modeling and targeting the bone niche as rigorously as the tumor cell itself. Historically, standard-of-care treatments have focused on tumor cell destruction or late-stage bone resorption, often failing to address the early colonization and dormant phases of metastasis (Satcher and Zhang, 2022). By viewing the bone microenvironment as an active participant—including its vascular and neural networks—new therapeutic strategies can be developed to disrupt the “soil” that nurtures metastatic “seeds” (Bakir et al., 2026a). Rigorous modeling of this environment is not merely a technical challenge but a clinical necessity to overcome resistance and improve patient survival. Taken together, these advances support a shift in how bone metastasis is studied and treated. The field must move beyond tumor-cell-centric models and therapies toward approaches that treat the bone lesion as a cellular, physical, chemical, vascular, immune, and neural ecosystem.

## 10 Conclusion

Bone metastases progression and therapy resistance cannot be fully explained by tumor-intrinsic mechanisms or cellular crosstalk alone. The metastatic bone lesion is a physically and chemically constrained ecosystem in which hypoxia, acidosis, mineralized matrix architecture, mechanical stress, impaired perfusion, interstitial pressure, and limited drug transport shape tumor survival and treatment response. These factors do not act in isolation; they interact with stromal, vascular, immune, neural, and bone-remodeling compartments to create distinct resistance states within the same disease process. Therefore, the next major step in the field is not simply to build more complex models, but to build more faithful and question-driven models. Platforms should be selected based on the lesion feature under study and validated against clinically meaningful endpoints. By aligning model design with niche-directed therapeutic strategies, the field can move toward better prediction of drug response, improved patient stratification, and more rational treatment combinations for bone metastatic disease.
